# Preoperative plasma fatty acid metabolites inform risk of prostate cancer progression and may be used for personalized patient stratification

**DOI:** 10.1186/s12885-019-6418-2

**Published:** 2019-12-16

**Authors:** Eugenio Zoni, Martina Minoli, Cédric Bovet, Anne Wehrhan, Salvatore Piscuoglio, Charlotte K. Y. Ng, Peter C. Gray, Martin Spahn, George N. Thalmann, Marianna Kruithof-de Julio

**Affiliations:** 1 Department for BioMedical Research, Urology Research Laboratory, University of Bern, Bern, Switzerland; 2 University Institute of Clinical Chemistry, Inselspital, Bern University Hospital, University of Bern, Bern, Switzerland; 3 Institute of Pathology, University Hospital Basel, University of Basel, Basel, Switzerland; 4 Visceral Surgery Research Laboratory, Clarunis, Department of Biomedicine, University of Basel, Basel, Switzerland; 5 Clarunis Universitäres Bauchzentrum Basel, Basel, Switzerland; 6 Department for BioMedical Research, Oncogenomics, University of Bern, Bern, Switzerland; 7 ScienceMedia Inc, 8910 University Center Ln Suite 400, San Diego, CA 92122 USA; 8 Zentrum für Urologie Zürich und Prostatakarzinomzentrum Hirslanden ZürichKlinik Hirslanden, Zürich, Switzerland; 9 Department of Urology, Essen University Hospital, University of Duisburg-Essen, Essen, Germany; 10 Department of Urology, Inselspital, Bern University Hospital, University of Bern, Bern, Switzerland

**Keywords:** Acylcarnitines, Prostate cancer, Metabolomics, Disease progression, Fatty acid metabolism

## Abstract

**Background:**

Little is known about the relationship between the metabolite profile of plasma from pre-operative prostate cancer (PCa) patients and the risk of PCa progression. In this study we investigated the association between pre-operative plasma metabolites and risk of biochemical-, local- and metastatic-recurrence, with the aim of improving patient stratification.

**Methods:**

We conducted a case-control study within a cohort of PCa patients recruited between 1996 and 2015. The age-matched primary cases (*n* = 33) were stratified in low risk, high risk without progression and high risk with progression as defined by the National Comprehensive Cancer Network. These samples were compared to metastatic (*n* = 9) and healthy controls (*n* = 10). The pre-operative plasma from primary cases and the plasma from metastatic patients and controls were assessed with untargeted metabolomics by LC-MS. The association between risk of progression and metabolite abundance was calculated using multivariate Cox proportional-hazard regression and the relationship between metabolites and outcome was calculated using median cut-off normalized values of metabolite abundance by Log-Rank test using the Kaplan Meier method.

**Results:**

Medium-chain acylcarnitines (C6-C12) were positively associated with the risk of PSA progression (*p* = 0.036, median cut-off) while long-chain acylcarnitines (C14-C16) were inversely associated with local (*p* = 0.034) and bone progression (*p* = 0.0033). In primary cases, medium-chain acylcarnitines were positively associated with suberic acid, which also correlated with the risk of PSA progression (*p* = 0.032, Log-Rank test). In the metastatic samples, this effect was consistent for hexanoylcarnitine, L.octanoylcarnitine and decanoylcarnitine. Medium-chain acylcarnitines and suberic acid displayed the same inverse association with tryptophan, while indoleacetic acid, a breakdown product of tryptophan metabolism was strongly associated with PSA (*p* = 0.0081, Log-Rank test) and lymph node progression (*p* = 0.025, Log-Rank test). These data were consistent with the increased expression of indoleamine 2,3 dioxygenase (IDO1) in metastatic versus primary samples (*p* = 0.014). Finally, functional experiments revealed a synergistic effect of long chain fatty acids in combination with dihydrotestosterone administration on the transcription of androgen responsive genes.

**Conclusions:**

This study strengthens the emerging link between fatty acid metabolism and PCa progression and suggests that measuring levels of medium- and long-chain acylcarnitines in pre-operative patient plasma may provide a basis for improving patient stratification.

## Background

In men, prostate cancer (PCa) is the most common type of cancer and the second leading cause of cancer-related death [1]. The majority of PCa patients have an indolent clinical course and can be managed with active surveillance or local treatment. However, for patients with aggressive metastatic disease that has failed hormonal therapy, available treatments currently only extend life by a few months [2]. To increase survival, it is crucial to improve patient stratification at diagnosis, as this would prevent overtreatment of indolent disease and place appropriate focus on patients with aggressive and potentially lethal tumors. Analysis of metabolites can highlight deviations from a particular physiologic status and metabolomics is already routinely employed in diagnostics to identify a variety of pathological situations [3, 4]. Metabolites can be measured in bodily fluids such as urine and plasma, which are readily accessible without the need for invasive procedures [5]. The application of metabolomics to PCa analysis has revealed that prostate tumors have metabolic profiles that are distinct from those of normal prostate tissues and other cancers [6–9]. PCa cells often use fatty acid (FA) metabolism as major source of energy production [10], while other cancer typically use glycolysis to sustain proliferation (Warburg Effect) [11, 12]. It has been proposed that de novo lipid biogenesis and β-oxidation are among the most altered pathways in PCa metabolism and PCa cells exhibit increased uptake of FAs, such as palmitate [13], as well as increased FA β-oxidation [14]. Additionally, PCa is frequently accompanied by the metabolic syndrome which presents altered blood lipid levels and obesity [15, 16]. Thyssle et al. [17] detected high levels of FAs in plasma of PCa patients with metastasis compared to patients without metastasis or with benign prostatic hyperplasia (BPH). Giskeødegård et al. also measured higher levels of plasma FAs (particularly acylcarnitines) in PCa patients compared to BPH controls [18]. Additionally, in a multi-center study [19], comparison of pre-diagnostic plasma from 1077 PCa cases and 1077 controls showed that long-chain acylcarnitines (C14:1, C18:1 and C18:2) were inversely associated with advanced PCa stages, while short-chain acylcarnitine C3 was positively associated with aggressive and lethal disease. Recently, the same authors confirmed these data in another cohort of 3057 matched case-control sets of pre-diagnostic plasma [20]. Crowe et al. [21] similarly found an association between palmitic acid and risk of low-grade PCa. These studies suggest that there is an inverse association between the precursors or early intermediates of FA β-oxidation (i.e. long-chain acylcarnitines) and risk of disease. Conversely, these findings suggest that shorter-chain acylcarnitines are positively correlated with aggressive disease.

In the present study we hypothesized that metabolic profiling of pre-operative plasma collected from individuals diagnosed with PCa will lead to the identification of biomarkers associated to disease progression and stratification. To test this hypothesis, we performed untargeted metabolomics analysis of pre-operative plasma from treatment naïve PCa patients who were classified as low risk, high risk without progression or high risk with progression, according to the PCa stratification criteria of the National Comprehensive Cancer Network (NCCN) [22]. In parallel, we analyzed plasma from PCa metastatic patients and healthy controls, in order to identify metabolites that could be specific for disease status and progression.

## Methods

### Study design and setting

We conducted a case-control study within a cohort of PCa patients who presented at the Department of Urology, Inselspital Bern University Hospital in Bern, Switzerland between 1996 and 2015. Primary PCa cases were aged between 46 and 74 years, did not present any apparent metastasis and received radical prostatectomy immediately after blood collection. Fasting blood was drawn early in the morning before surgery, processed and separated into components [23]. Metastatic cases were aged between 54 and 86 years at time of blood collection and already presented metastasis. Healthy controls were aged between 29 and 55 at blood collection. For all participants, the plasma was collected in EDTA tubes and stored at − 80 °C. All participants provided informed consent for participation in this study.

### Cases and controls

Primary cases were men who received a PCa diagnosis following digital rectal examination, prostate specific antigen (PSA) assessment and TNM staging evaluation of prostate biopsies prior to blood collection. The primary cases (*n* = 33) were selected in order to identify at least 10 age matched patients for each of the PCa stratification levels defined according to the National Comprehensive Cancer Network (NCCN) [24, 25] and to integrate these with clinical follow-up, namely low risk (T1-T2a, Gleason score ≤ 6, PSA < 10 ng/mL), high risk without progression (T ≥ 3a, Gleason score 8–10, PSA > 20 ng/mL, without local or bone progression within 5 years after radical prostatectomy) and high risk with progression (T ≥ 3a, Gleason score 8–10, PSA > 20 ng/mL with local or distant progression at last follow-up within 5 years from radical prostatectomy). PSA progression was defined as two consecutive rising PSA measurements > 0.1 ng/mL. Local progression was confirmed by imaging modality and/or biopsy. Lymph node progression was identified by imaging modalities (MRI, CT-scan or Choline/PSMA-PET CT). Bone progression was identified by skeletal scintigraphy. Of all the primary PCa patients (*N* = 33), only one (*N* = 1) received androgen deprivation therapy (Casodex) prior to blood collection.

Metastatic cases were men (*n* = 9) diagnosed with PCa and who presented evidence of metastatic spreading as documented by skeletal scintigraphy. These are advanced patients, who already received either radiation therapy and/or androgen deprivation treatments. The blood collected from these individuals was not fasting. Controls were healthy individuals (*n* = 10) with no evidence of disease, who volunteered to participate in this pilot study.

### Untargeted metabolomics analysis

Plasma samples were extracted and analyzed by reversed-phase chromatography coupled to high-resolution mass spectrometry (HRMS) as previously described [26]. Metabolic features measured by UHPLC-HRMS were isolated with Progenesis QI (version 2.2, Nonlinear Dynamics, Newcastle, UK) and analyzed as previously described [26]. Potential structures and formulas of the isolated metabolic features were searched against the Human Metabolome Database (HMDB, version 3.6) [27] with a mass accuracy of 8 ppm and against an in-house database containing retention times of a set of 378 compounds. Features identified against the in-house database with a retention time deviation < 0.4 min were accepted as potential identity. The assigned features were manually reviewed for correct peak shape and identity assignment with MS/MS data.

### Statistical analysis

Hazard ratios (HRs) for the association between risk of progression and normalized abundances by median cut-off based on the distribution in primary cases were calculated using multivariate Cox proportional-hazard regression model using the Survival [28, 29] and Survminer [30] R package. Normalized abundances data were used to estimate the associations and data were tested for proportional-hazards (PH) assumption and by graphical diagnostics based on the scaled Schoenfeld residuals [31] prior to analysis. The corresponding HRs represent the risk associated with a higher or lower (median cut-off) abundance of the indicated metabolite. Kaplan Meier curves to illustrate the progression of the stratified risk groups in primary PCa cases were calculated using the “survfit” function and the Log-Rank test using the Survival R package [28, 29]. Kaplan Meier curves to identify the association between selected metabolites and patient outcome were calculated using median cut-off normalized values of metabolites abundances with the “survfit” function and differences estimated with the Log-Rank test with the Survival R package [28, 29]. Correlation among the metabolites were calculated using the Pearson parametric correlation test (cut-off *p* < 0.05) with the Hmisc R package [32]. Differential expression analysis on publically available dataset was conducted with ShinyGEO [33]. Visualization of genomic data was generated with cBioPortal [34, 35]. For calculation of Kaplan Meier curves, TCGA PRAD gene expression data were retrieved from Firehose [36] with RTCGAToolbox R package [37]. Data were downloaded as RSEM normalized values and transformed to log2 in order to achieve normal distribution, genes whose expression was 0 in more than 50% of the samples were removed from analysis. If more than one follow-up was available data were collapsed and the higher information (more recent) maintained. Survival was estimated by calculation of optimal cut points using the Survminer R package [30] and differences identified by Log-Rank test. Principal component analysis (PCA) was conducted on the normalized abundances of the m/z features measured in positive and negative mode by applying log2 transformation and data scaling with the FactoMineR R package [38]. Features with abundances equal to 0 in more than 50% of the sample were excluded from the analysis. Additionally, abundances lower than 250 were removed to reduce noise. Eigenvalues were used to determine the number of principal components to be considered and to display the variability of the data. Confidence interval corresponding to 95% is represented in the ellipses on the PCA plots. For all the data representation, graphical plots were generated using the ggplot2 R package [39]. The analyses were done using RStudio version 1.1.463 [40] and R version 3.5.3 [41].

### Cell lines and culture conditions

The parental cell lines used in this study have been authenticated using highly polymorphic short tandem repeat (STR) loci. PC-3 M-Pro4 cells originate from serial passage of PC-3 M cells in the prostate of athymic mice [42] and were cultured in DMEM (GibcoBRL) with 4.5 g glucose/L, 10% FCII (Thermo Fisher Scientific), 1% penicillin–streptomycin (PS, Life Technologies). C4–2 [43], C4–2B4 [44] and LNCaP [45] cells were cultured in T-medium DMEM (Sigma-Aldrich) with 20% F-12 K nutrient mixture Kaighn’s modification (GibcoBRL), 10% FCS, 0.125 mg/mL biotin, 1% insulin-transferrin-selenium (ITS), 6.825 ng/mL T3, 12.5 mg/mL adenine, 1% PS. LNCaP cells were derived from PCa lymph node metastasis [46], C4–2 cells was derived from LNCaP cells by passaging in castrated mice [43] and C4–2B from a bone metastasis of LNCaP tumor in nude mice [44]. All cells were cultured at 37 °C and 5% CO2.

### Cell proliferation assay

To assess proliferation, cells were seeded at a density of 1500 cells per well and growth monitored for 48 h. AU 490 nm was measured 2 h after incubation with 20 μL of 3-(4,5 dimethylthiazol- 2-yl)- 5 -(3 -carboxymethoxyphenyl)- 2 -(4 -sulfophenyl)- 2 Htetrazolium (MTS, Promega) at 37 °C according to manufacturer’s protocol. Data were normalized for the number of cells seeded and fold change estimated versus control condition (vehicle treatment).

### Quantitative real-time PCR (qPCR) analysis

Total RNA was extracted using Trizol (Invitrogen) and cDNA synthesized according to manufacturer’s instructions (Promega) and as previously described [47]. Real-time qPCR was performed with QuantStudio3 (Thermo Fisher Scientific). HPRT was used for normalization and relative expression is calculated with 2^−ΔCt^ formula. Primers sequences are indicated in Additional file 1: Table S1.

### Dihydrotestosterone (DHT) and palmitic acid (PA) stimulation

DHT was diluted in ethanol (EtOH) and administered at a final concentration of 10 nmol/L [48]. EtOH was used as control. PA was prepared in albumin fatty acid free (BSA-FFA) [49] (Sigma) and administered at a final concentration of 100 μM [50]. BSA-FFA was used as control. For both treatments, incubation time was 48 h [50]. Before the treatment, in order to wash out the androgens, cells were starved for 48 h in medium containing charcoal stripped serum (CSS). Experimental conditions were replicated (*n* = 3, biological repeats) and measured (*n* = 2, technical replicate for each sample).

## Results

### Characterization of cases and controls

**Fig. 1:**
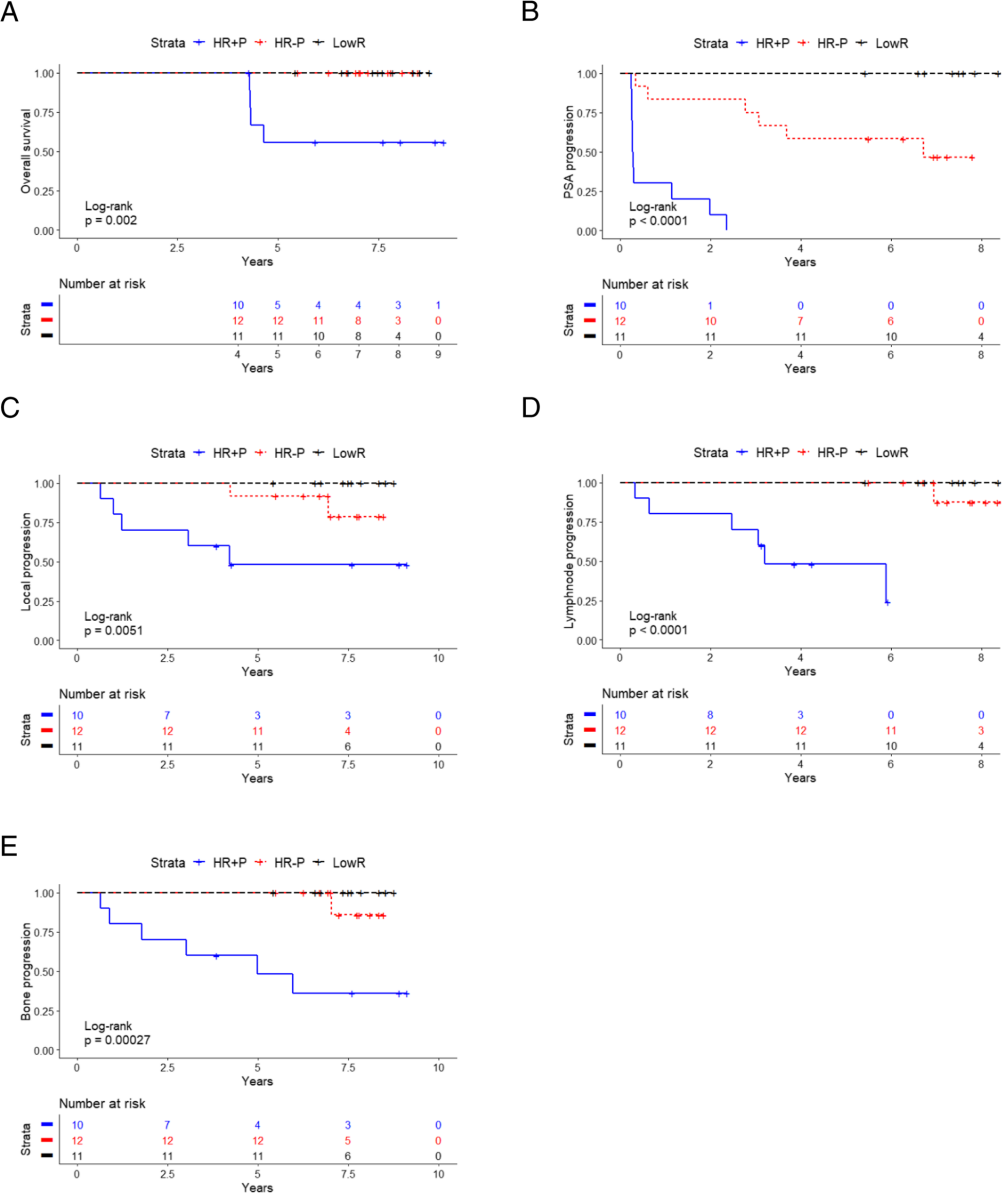
Characterization of disease progression in PCa cases. **a** Overall survival calculated by the Kaplan Meier method for high-risk with progression (HR + P, blue solid line), high-risk without progression (HR-P, red dotted line) and low-risk (LR, black dashed line) PCa cases. *P*-value estimated with Log-Rank test. **b** PSA progression free survival calculated by the Kaplan Meier ethod for HR + P, HR-P and LR PCa cases. *P*-value estimated with Log-Rank test. **c** Local progression free survival calculated by the Kaplan Meier method for HR + P, HR-P and LR PCa cases. *P*-value estimated by the Log-Rank test. **d** Lymph node progression-free survival calculated by the Kaplan Meier method for HR + P, HR-P and LR PCa cases. P-value estimated by the Log-Rank test. **e** Bone progression-free survival calculated by the Kaplan Meier method for HR + P, HR-P and LR PCa cases. P-value estimated with the Log-Rank test. For statistical details see Methods section

Characteristics of all samples (42 cases and 10 controls) and subgroups of PCa risk categories as classified in the NCCN guidelines (v1.2018) [24] (11 low risk cases, 12 high risk without progression, 10 high risk with progression and 9 Metastatic) are shown in Table 1. All individuals included in the study were Caucasian. The median age of the patients at blood draw was 61.4 years for the low risk group, 62.9 for the high risk without progression group, 63.6 for high risk with progression group and 59.8 for the metastatic group, while the median age of the healthy controls was 38.3 years at blood draw. The PSA median level at blood draw was 5.8 ng/mL for low risk patients, 9.32 ng/mL for high risk patients without progression and 10.9 ng/mL for high risk patients with progression. A median follow-up higher than 5 years (after radical prostatectomy) is available for all the primary cases (7.59 years for the low risk, 7.14 for the high risk without progression and 5.11 for high risk with progression). For the metastatic cases the median follow-up is 3.23 years from blood draw. Among the primary cases, high risk with progression had a significantly lower overall survival compared to low risk and high risk without progression (Log-Rank *p* = 0.002) (Fig. 1a). Both high risk patients without local and bone progression and high risk patients with local and bone progression experienced PSA progression (following radical prostatectomy) at a median of 5.88 years and 0.28 years, respectively, compared to low risk (no biochemical relapse, log-rank *p* < 0.0001) (Fig. 1b). High risk patients with progression displayed local recurrence at a median of 4.04 years after radical prostatectomy while high risk patients without progression had local recurrence at 6.98 years compared to low risk cases where no progression was observed (log-rank *p* = 0.0051) (Fig. 1c). Lymph node progression and bone progression were the two main events discriminating the high and low risk groups. Lymph node progression occurred at a median of 3.16 years in the high risk with progression cases while for high risk without progression group the median was essentially comparable to low risk cases (7.14 versus 7.59 years, respectively, after radical prostatectomy) (Log-Rank *p* < 0.0001) (Fig. 1d). Bone progression was detected in high risk with progression cases at a median of 5.11 years after radical prostatectomy compared to 7.14 and 7.59 years for high risk without progression and low risk, respectively (Log-Rank *p* = 0.00027) (Fig. 1e).

**Table 1:** Characteristics of the cases and controls

Characteristics	Cases				Controls
	Low Risk N (%)	High Risk without Progression N (%)	High Risk with progression N (%)	Metastatic N (%)	Healthy N (%)
Number of subjects	11 (100)	12 (100)	10 (100)	9 (100)	10 (100)
Ethnicity					
Caucasian	11 (100)	12 (100)	10 (100)	9 (100)	10 (100)
	Median (IQR)	Median (IQR)	Median (IQR)	Median (IQR)	Median (IQR)
Age at blood draw (years)	61.4 (57.6–64.6)	62.9 (60.2–69.3)	63.6 (60.6–68.3)	59.8 (57–68)	38.3 (34–40.6)
PSA at blood draw (ng/mL)	5.8 (4.6–6.05)	9.32 (6.12–13.6)	10.9 (7.65–23.9)	NA^a^	NA^b^
Years between blood draw and event					
PSA progression (or last follow-up)	7.59 (7.05–8.44)	5.88 (2.99–6.96)	0.28 (0.26–0.93)	NA^a^	NA^b^
local progression (or last follow-up)	7.59 (7.05–8.44)	6.98 (6.60–7.76)	4.04 (1.69–6.77)	NA^a^	NA^b^
lymphnode progression (or last follow-up)	7.59 (7.05–8.44)	7.13 (6.88–7.87)	3.16 (2.62–4.14)	NA^a^	NA^b^
bone progression (or last follow-up)	7.59 (7.05–8.44)	7.14 (6.88–7.87)	4.42 (2.09–7.20)	NA^a^	NA^b^
Years in follow-up	7.59 (7.05–8.44)	7.14 (6.88–7.87)	5.11 (4.25–7.94)	3.23 (1.77–4.87)	NA^b^

Prostate cancer patient classification is determined according to *NCCN* guidelines version 1.2018; *IQR*, interquartile range (25th to 75th percentile)

^a^ The metastatic patients have not received radical prostatectomy and already present with advanced metastatic and progressive prostate cancer at time of blood draw

^b^ The controls are healthy and do not present prostate cancer

### Metabolomics analysis

**Fig. 2:**
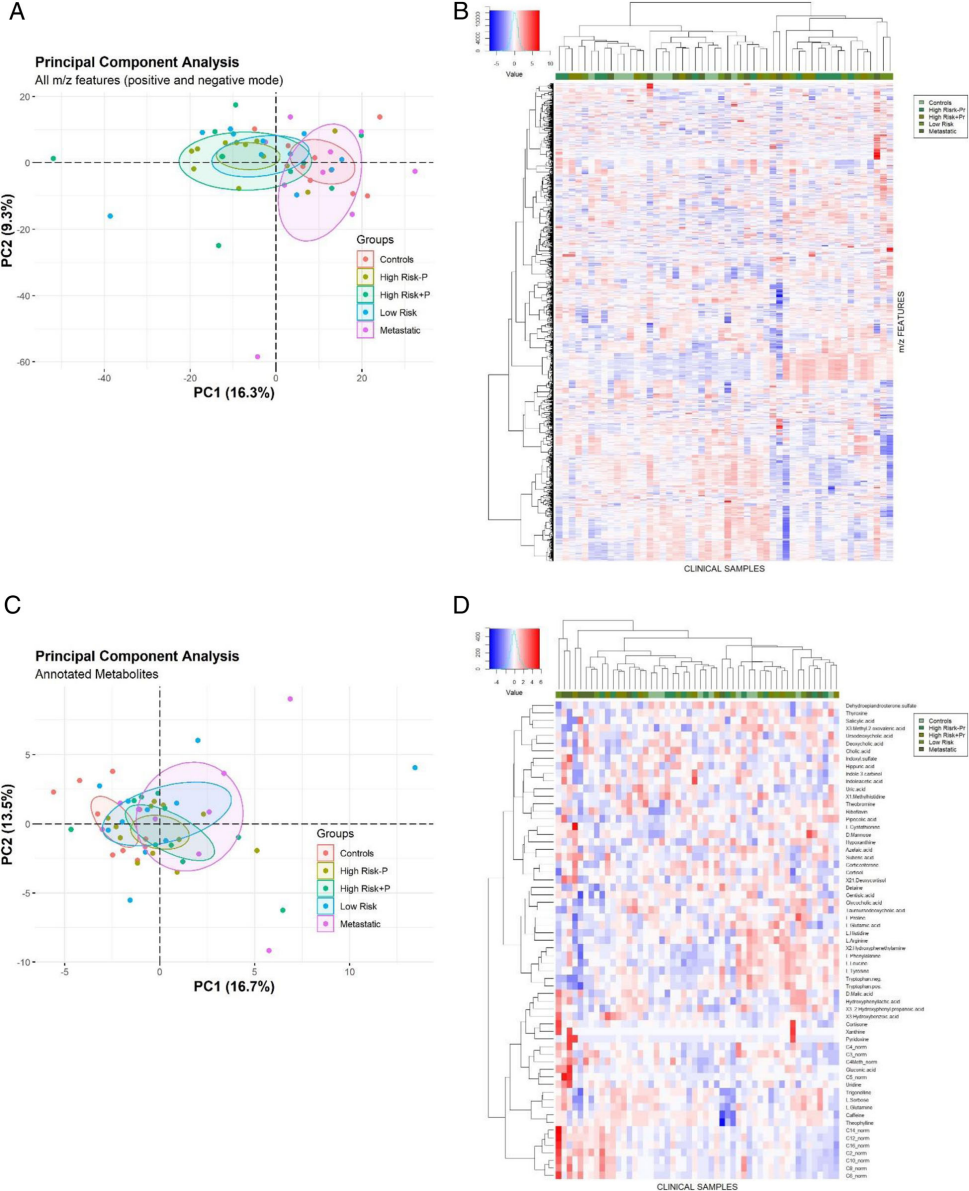
Sample classification by principal component analysis and overview of metabolomics profiling. **a** Principal component analysis (PCA) of all the m/z features measured in positive and negative ionization mode. The variation retained by PC1 (16.3%) is represented of the X axis and the variation retained by the PC2 (9.3%) is represented on the Y axes. Ellipses represent the 95% confidence interval for each group. **b** Heatmap generated with scale and centered normalized m/z abundances measured in positive and negative ionization mode. Euclideian distance between groups and Minkowski distance between metabolites was used and clustering was calculated with the Ward (Ward.D2) method for minimum variance between m/z values. **c** Principal component analysis (PCA) of the annotated m/z features in positive and negative ionization mode. The variation retained by PC1 (16.7%) is represented of the X axis and the variation retained by the PC2 (13.5%) is represented on the Y axis. Ellipses represent the 95% confidence interval for each group. **d** Heat map generated with scale and centered normalized m/z abundances of the annotated metabolites. Same methods used for Fig. 2b were applied. For statistical details see Methods section

Principal component analysis of all the m/z features measured in positive and negative ionization mode was able to distinguish primary versus metastatic cases and healthy controls (Fig. 2a and Additional file 2: Figure S1A). However, a heatmap of all the m/z features demonstrated that hierarchical clustering could not differentiate between the primary subgroups (low risk versus high risk without progression versus high risk with progression) (Fig. 2b).

Principal component analysis of the annotated metabolites was able to distinguish healthy controls versus patients, but not between the primary risk subgroups (Fig. 2c, Additional file 2: Figure S1B and Fig. 2d).

### Characterization of acylcarnitines distribution and prognostic associations

**Fig. 3:**
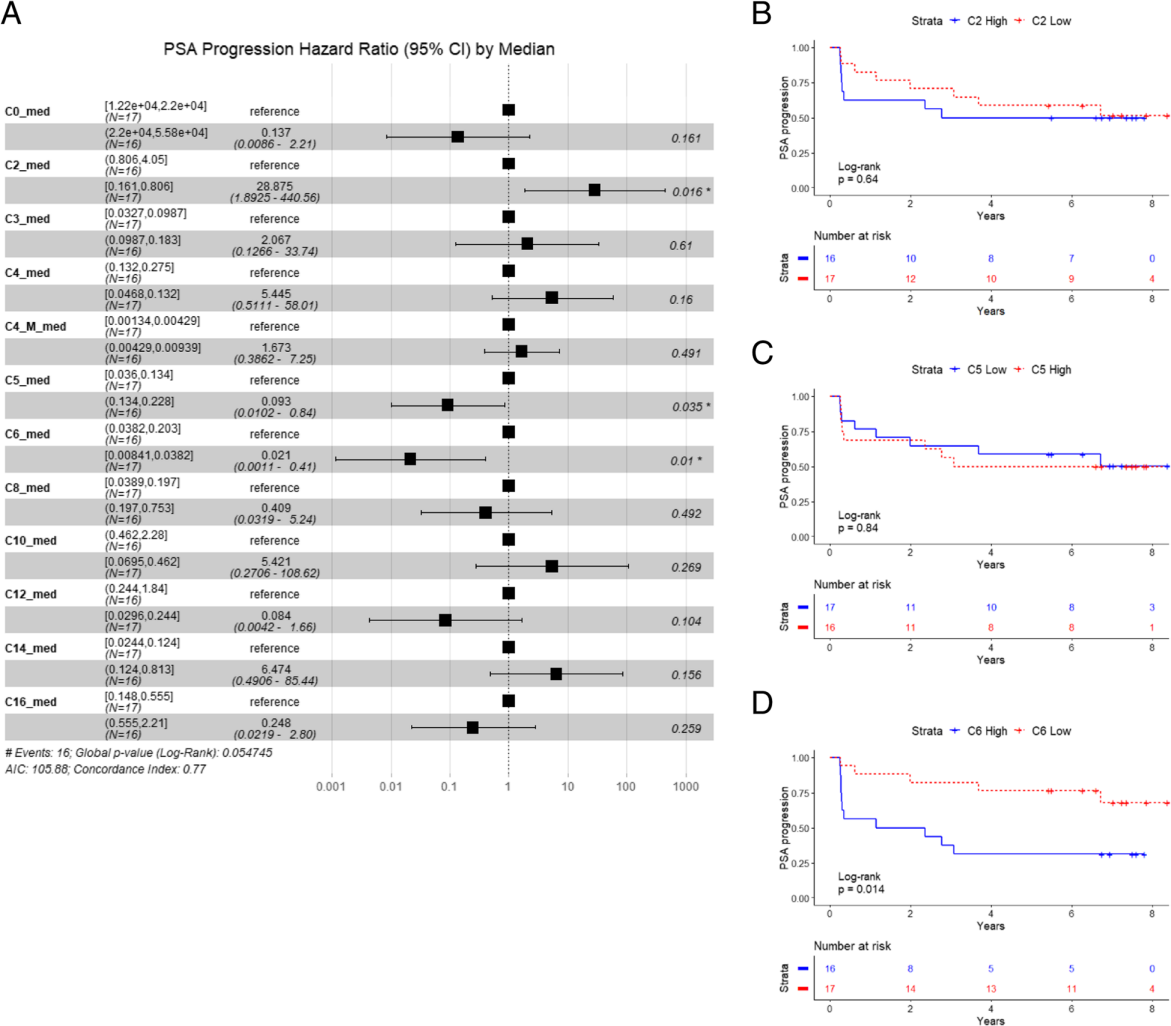
Hazard ratio (95% confidence interval) and Kaplan Meier curves for the association of acylcarnitines with PSA progression. **a** Hazard ratios and 95% confidence interval (CI) for the association of the complete panel of acylcarnitines with the risk of PSA progression. Groups (lower and higher risk) were separate by median cut-off (suffix “_med”) of the normalized abundances for each molecule. C4 = butyrylcarnitine, C4_M = methylmalonylcarnitine. **b** PSA progression-free survival calculated by the Kaplan Meier method for acetylcarnitine (C2). Groups are defined by median-cut off of normalized abundances (vs L-carnitine) of high acetylcarnitine (C2 High, blue solid line) vs low acetylcarnitne (C2 Low, red dotted line). *P*-value was estimated with Log-Rank test. **c** PSA progression free survival calculated by the Kaplan Meier method for isovalerylcarnitine (C5). Groups are defined by median-cut off of normalized abundances (versus L-carnitine) of High isovalerylcarnitine (C5 High, red dot line) versus low isovelrylcarnitne (C5 Low, blue solid line). *P*-value was estimated with Log-Rank test. **d** PSA progression-free survival calculated by the Kaplan Meier method for hexanoylcarnitine (C6). Groups are defined by median-cut off of normalized abundances (vs L-carnitine) of high hexanoylcarnitine (C6 High, blue solid line) versus low hexanoylcarnitne (C6 Low, red dotted line). P-value was estimated with Log-Rank test. For statistical details see Methods section

A complete panel of acylcarnitines (from C2 to C16) was detected in patients and controls (Table 2, values normalized against L-carnitine (C0) levels). We analyzed the impact of acylcarnitines on disease associated risks by multivariate Cox regression models, for all the primary patients, given that the median age of the individuals in these groups was highly comparable. Medium- chain acylcarnitines (C6-C12) were positively associated with risk of PSA progression (*p* = 0.036, median cut-off) (Additional file 3: Figure S2A) and long-chain acylcarnitines were inversely associated with local and bone progression risk (*p* = 0.034 and *p* = 0.033 respectively, median cut-off) (Additional file 3: Figure S2B and S2D). No association with lymph node progression risk was detected by short-, medium-, or long-chain acylcarnitines (using pooled values for class of molecules, namely C2-C5 (short-chain), C6-C12 (medium-chain), C14-C16 (long-chain), normalized against C0, median cut-off) (Additional file 3: Figure S2C). When we investigated the contribution of the single molecules, we found an inverse association between acetylcarnitine (C2) and risk of PSA progression (*p* = 0.016, global Log-Rank *p* = 0.05 with multivariate Cox regression analysis for the panel of acylcarnitine associated with risk of PSA progression tested for proportional hazard assumption, median cut-off) (Fig. 3a). Isovalerylcarnitine (C5) and hexanoylcarnitine (C6) were inversely and positively respectively associated with risk of PSA progression (*p* = 0.035 and p = 0.01, respectively, normalized against C0, median cut-off) (Fig. 3a). However, calculation of Kaplan-Meier curves for PSA progression (using Log-Rank test), did not display a significant difference for C2 and C5 (High versus Low, median cut-off), while higher C6 was significantly associated with PSA progression (Log-Rank *p* = 0.014) (Fig. 3b, c and d).

**Table 2:** Geometric mean (95% confidence interval) normalized abundances of acylcarnitines in Cases and Controls

	Cases				Controls
Aylcarnitines (normalized abundance)	Low Risk	High Risk without Progression	High Risk with progression	Metastatic	Healthy
L.Carnitine (C0)	22,341 (17389–28,703)	24,578 (20774–29,078)	22,514 (17877–28,355)	23,767 (21161–26,695)	25,038 (19910–31,487)
Total Carnitines	87,554 (71512–107,194)	95,564 (80237–113,817)	88,145 (78007–99,600)	106,009 (77213–145,545)	77,690 (65760–91,784)
Total Carnitines (norm.)	2.81 (1.89–4.18)	2.80 (2.04–3.84)	2.80 (1.89–4.14)	3.36 (2.26–5.00)	2.07 (1.68–2.55)
Short Chain Acylcarnitines (C2-C5 norm.)	1.24 (0.84–1.83)	1.12 (0.82–1.52)	1.19 (0.82–1.72)	1.65 (1.08–2.51)	0.93 (0.76–1.13)
Medium Chain Acylcarnitines (C6-C12 norm.)	0.87 (0.55–1.39)	1.00 (0.65–1.54)	0.93 (0.55–1.57)	0.93 (0.52–1.64)	0.52 (0.34–0.80)
Long Chain Acylcarnitines (C14-C16 norm.)	0.70 (0.48–1.03)	0.67 (0.53–0.84)	0.65 (0.45–0.95)	0.71 (0.55–0.93)	0.56 (0.45–0.69)
Acylcarnitine (C2 norm.)	0.83 (0.55–1.25)	0.75 (0.52–1.06)	0.79 (0.50–1.23)	0.95 (0.66–1.37)	0.59 (0.48–0.72)
Propionylcarnitine (C3 norm.)	0.09 (0.06–0.14)	0.07 (0.05–0.10)	0.09 (0.07–0.12)	0.10 (0.07–0.14)	0.09 (0.06–0.13)
Butyrylcarnitine (C4 norm.)	0.11 (0.06–0.18)	0.10 (0.07–0.13)	0.12 (0.09–0.15)	0.14 (0.09–0.23)	0.11 (0.06–0.18)
Methylmalonylcarnitine (C4 Methyl norm.)	0.004 (0.003–0.005)	0.003 (0.002–0.004)	0.004 (0.003–0.006)	0.005 (0.003–0.008)	0.003 (0.002–0.004)
Isovalerylcarnitine (C5 norm.)	0.12 (0.08–0.18)	0.11 (0.08–0.14)	0.12 (0.09–0.15)	0.18 (0.06–0.52)	0.09 (0.07–0.13)
Hexanoylcarnitine (C6 norm.)	0.03 (0.02–0.05)	0.03 (0.02–0.05)	0.04 (0.02–0.06)	0.04 (0.02–0.07)	0.02 (0.01–0.03)
L.Octanoylcarnitine (C8 norm.)	0.17 (0.12–0.26)	0.22 (0.14–0.34) ^a^	0.20 (0.12–0.32)	0.20 (0.11–0.37)	0.11 (0.07–0.17)
Decanoylcarnitine (C10 norm.)	0.41 (0.26–0.66)	0.50 (0.32–0.78) ^a^	0.45 (0.26–0.77)	0.44 (0.25–0.80)	0.25 (0.15–0.40)
Dodecanoylcarnitine (C12 norm.)	0.23 (0.13–0.39)	0.23 (0.15–0.35) ^a^	0.23 (0.13–0.40)	0.22 (0.12–0.37) ^a^	0.13 (0.09–0.19)
Tetradecanoylcarnitine (C14 norm.)	0.13 (0.08–0.211)	0.12 (0.09–0.17)	0.12 (0.07–0.19)	0.13 (0.09–0.20)	0.09 (0.07–0.11)
L.Palmitoylcarnitine (C16 norm.)	0.56 (0.44–0.74)	0.53 (0.43–0.67)	0.53 (0.37–0.75)	0.57 (0.44–0.74)	0.46 (0.37–0.57)

Acylcarnitines abundance is normalized by data mean intensities and by *C0* (L.Carnitine) abundance (norm.). ^a^ *OPLS-DA* models selected based on multivariate statistical analysis (Controls vs Specific Cases)

**Fig. 4:**
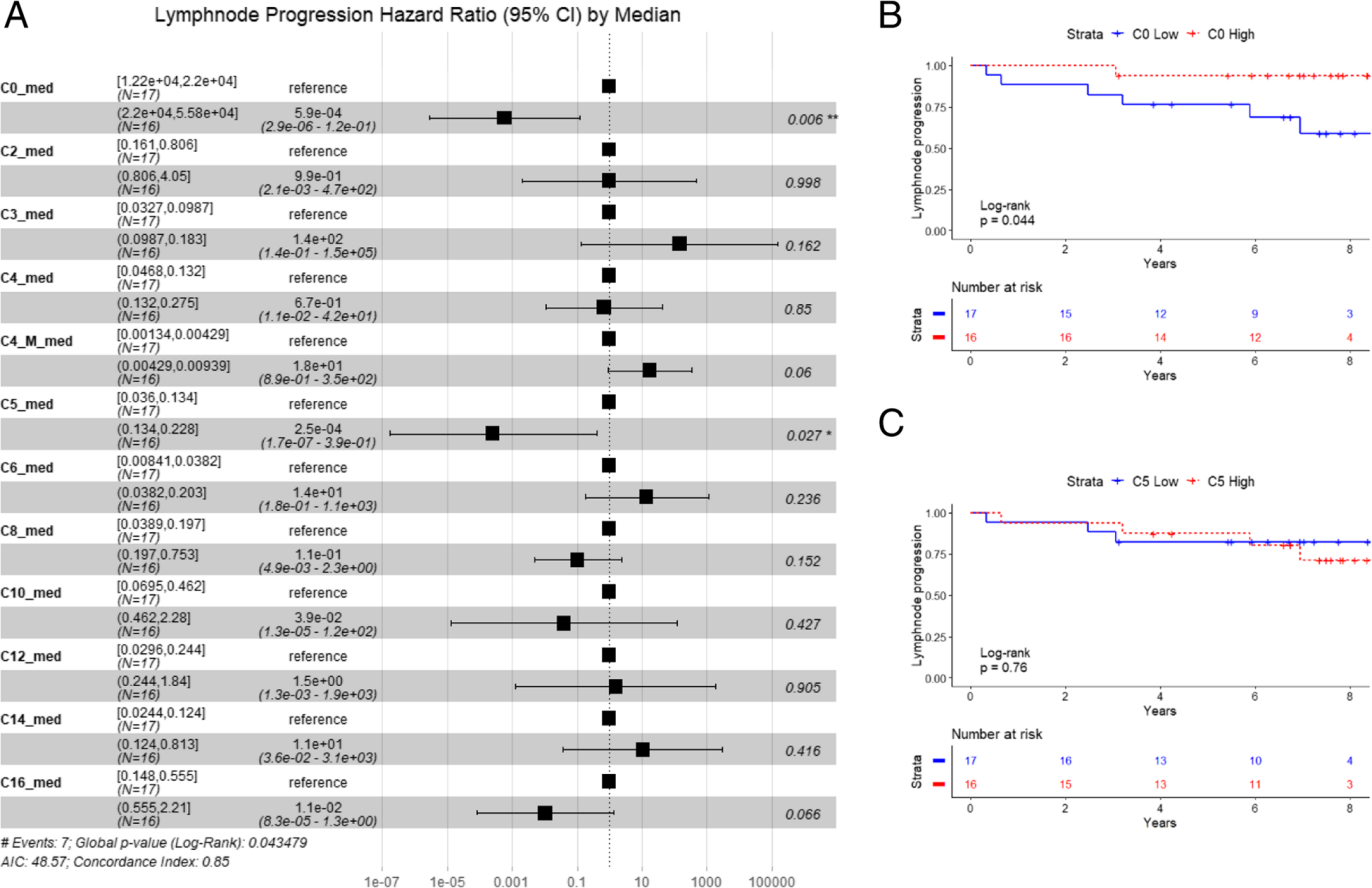
Hazard ratio (95% confidence interval) and Kaplan Meier curves for the association of acylcarnitines with lymph node progression. **a** Hazard ratios and 95% confidence interval (CI) for the association of the complete panel of acylcarnitines with the risk of lymph node progression. Groups (lower and higher risk) were separate by median cut-off (suffix “_med”) of the normalized abundances for each molecule. C4 = butyrylcarnitine, C4_M = methylmalonylcarnitine. **b** Lymph node progression-free survival calculated by the Kaplan Meier method for L-carnitine (C0). Groups are defined by median-cut off of normalized abundances of high L-carnitine (C0 High, red dotted line) versus low L-carnitne (C0 Low, blue solid line). *P*-value was estimated with Log-Rank test. **c** Lymph node progression free survival calculated by the Kaplan Meier method for isovalerylcarnitine (C5). Groups are defined by median-cut off of normalized abundances (versus L-carnitine) of high isovalerylcarnitine (C5 High, red dotted line) vs low isovelrylcarnitne (C5 Low, blue solid line). *P*-value was estimated with Log-Rank test. For statistical details see Methods section

While no association with bone progression was established, we found an inverse association between L-carnitine (C0) and isovalerylcarnitine (C5) and risk of lymph node progression (*p* = 0.006 and *p* = 0.027, respectively, median cut-off, Log-Rank *p* = 0.04 with multivariate Cox regression analysis) (Fig. 4a). Calculation of Kaplan-Meier curves for lymph node progression displayed a significant difference for C0 (High versus Low, median cut-off, log-rank *p* = 0.044), but not for C5. (Fig. 4b and c). We did not find a significant association between the classes of acylcarnitines and the risk of bone progression.

**Fig. 5:**
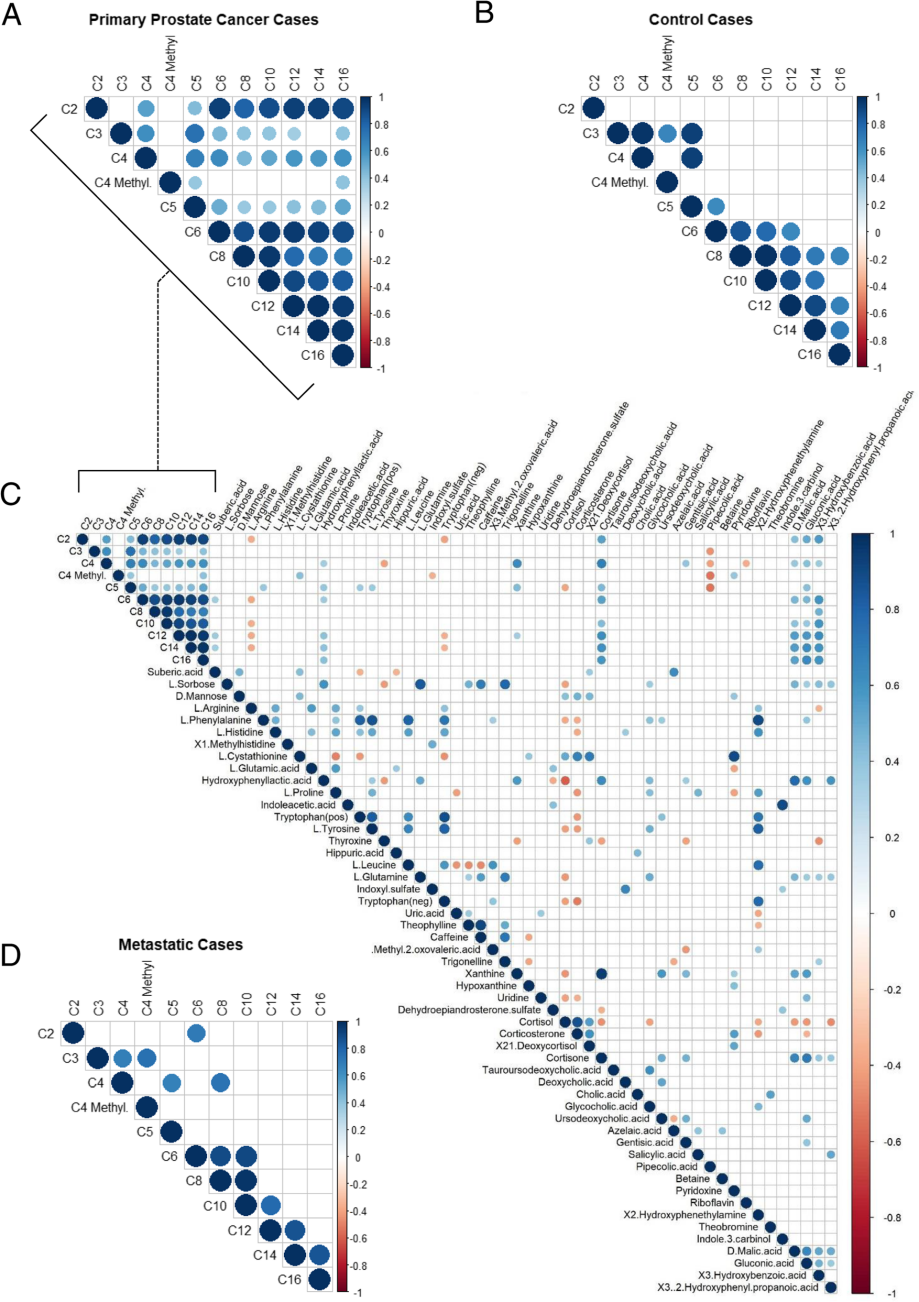
Correlation matrix for all metabolites identified in PCa cases and controls. Correlations are shown for the different acylcarnitines in primary PCa cases (**a**) and controls (**b**). **c** Insert with plot A displays the correlation among the acylcarnitines and all the annotated metabolites in primary cases. **d** Correlation among acylcarnitines in metastatic cases. The sizes of the circles are dependent on the Pearson correlation coefficient. Blue circles correspond to positive correlations and red circles correspond to negative correlations. Correlations that do not reach significance (*p* > 0.05) are indicated by an empty square box. For statistical details see methods section

As depicted in Fig. 5a we found a strongly and positive correlation between C2 and medium- (C6-C12) and long-chain (C14-C16) acylcarnitines in primary cases (cut-off *p* < 0.05), compared to control cases (Fig. 5b). We also detected a strong and positive correlation between the classes of medium- and long-chain acylcarnitines in primary and control cases, compared to metastatic cases (Fig. 5d).

### Suberic acid and indoleacetic acid are associated with PSA progression

**Fig. 6:**
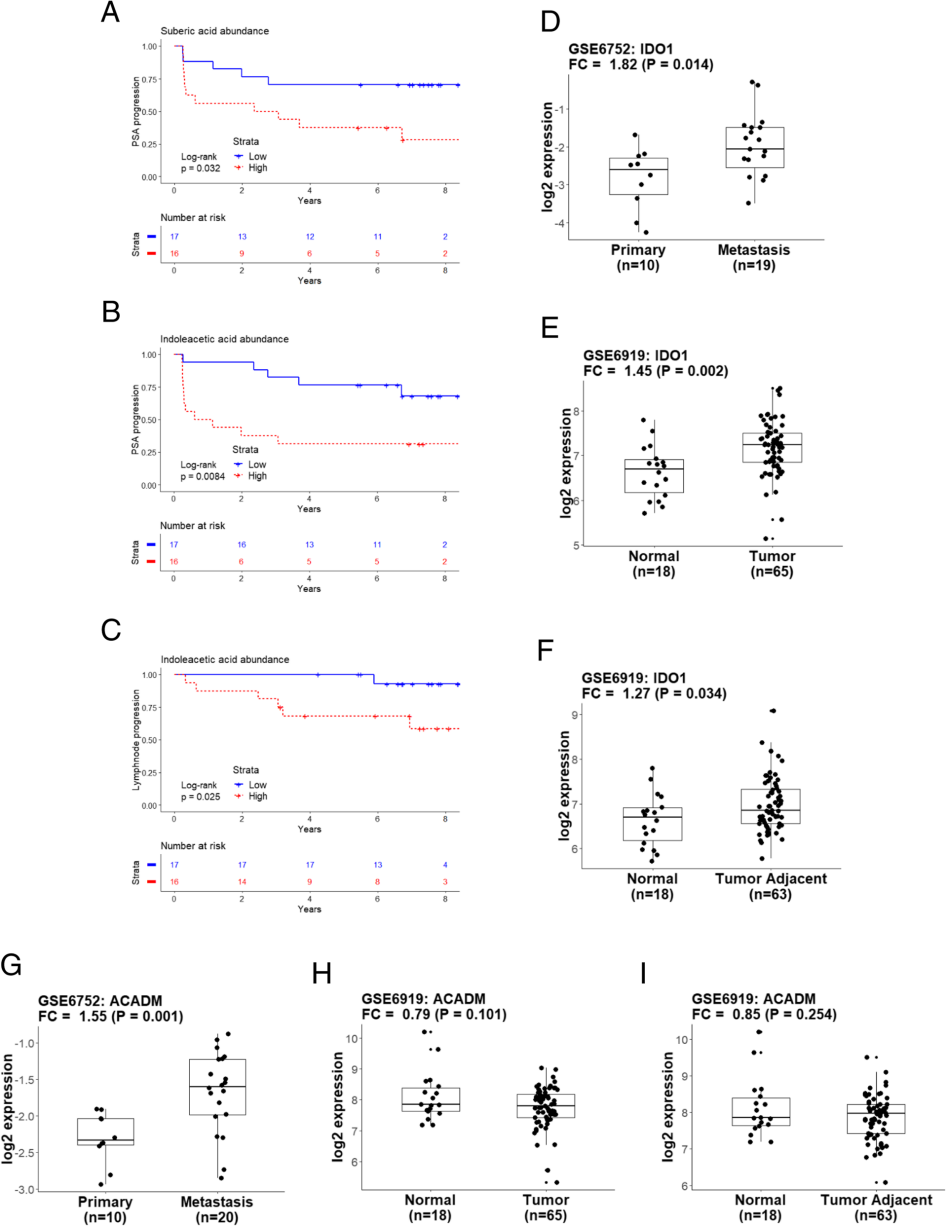
Association between annotated metabolites and disease progression and analysis of gene expression data. PSA progression-free survival calculated by the Kaplan Meier method for (**a**) suberic acid and (**b**) indoleacetic acid and lymph node progression-free survival for indoleacetic acid is represented in (**c**). Groups are defined by median-cut off of normalized abundances (low abundance, blue solid line) vs high abundance (red dotted line). *P*-value was estimated with log-rank test. *IDO1* expression data of primary and androgen ablation resistant metastasis are shown from GSE6752 (**d**) and GSE6919 (**e**-**f**). *ACADM* expression data in the same set of samples are displayed in (**g**-**h**-**i**). Fold change (FC) is calculated versus the normal or primary tumor samples and *p*-value (P) for significance between two groups estimated by t-test. For statistical details see Methods section

When the analysis was extended to all the annotated metabolites, we found a positive and significant correlation in primary patients between medium- and long-chain acylcarnitines (namely C6, C12 and C14) and suberic acid, an unsaturated dicarboxylic acid that is frequently elevated in patients with fatty acid oxidation disorders [51] (Fig. 5c). This association was stronger in the metastatic cases, for hexanoylcarnitine (C6), L-octanoylcarnitine (C8) and decanoylcarnitine (C10) (all belonging to the medium-chain acylcarnitines class) (Additional file 4: Figure S3). In the metastatic cases we also detected a remarkable strong and significant inverse correlation between medium-chain acylcarnitines and tryptophan detected in positive and negative ionization mode (this effect was consistent also in primary cases). When we calculated the Kaplan-Meier curves (Log-Rank test by median cut-off), we found that high levels of suberic acid were significantly associated with PSA progression (*p* = 0.032, Log-Rank test) (Fig. 6a). Furthermore, high levels of indoleacetic acid, a breakdown product of tryptophan metabolism directly downstream indoleacetaldehyde, were significantly associated with PSA and lymph node progression (*p* = 0.0081 and *p* = 0.025, respectively, Log-Rank test) (Fig. 6b and c). Notably, indoleacetaldehyde is metabolized to indoleacetic acid by aldehyde dehydrogenase 7 family member A1 (ALDH7A1), which is associated with aggressive PCa [52, 53]. All these assigned features were manually reviewed for correct peak shape and identity assignment with MS/MS data (Additional file 5: Table S2).

### Indoleamine 2,3-dioxygenase (IDO1) and medium-chain acyl-CoA dehydrogenase (*ACADM*) are elevated in metastatic prostate cancer

Indoleamine 2,3-dioxygenase (IDO1) was recently shown to enhance carnitine palmitoyltransferase I (CPT1) activity and fatty acid oxidation by degrading L-tryptophan [54]. This prompted us to search for a possible association between IDO1 and the medium-chain acylcarnitines pattern that we detected in primary and metastatic cases. To do this, we investigated the expression of *IDO1* and medium-chain Acyl-CoA Dehydrogenase (*ACADM*) in primary and metastatic PCa by analyzing gene expression profiles from datasets containing androgen-ablation resistant PCa metastatic samples [55] and normal and tumor adjacent tissues [55, 56]. We found a significant increase of *IDO1* in metastasis VS primary PCa (*p* = 0.014) (Fig. 6d and Additional file 6: Figure S4A) and a similar upregulation was detected in tumor and tumor adjacent tissues compared to normal tissue (Fig. 6e, f and Additional file 6: Figure S4B). While *ACADM* was similarly higher in primary PCa tissue compared to metastasis tissue (*p* = 0.001) (Fig. 6g and Additional file 6: Figure S4C), there was no difference in *ACADM* expression in tumor tissue compared to tumor adjacent tissues (Fig. 6h, i and Additional file 6: Figure S4D). When we looked at the distribution of *IDO1* and *ACADM* genomic alteration in a panel of PCa studies, we found a substantial distribution of deletions and amplifications of *IDO1* while *ACADM* also appeared to be mutated in a considerable fraction of the samples (Additional file 7: Figure S5A and S5B). However, higher transcriptional levels of IDO1 were not associated with disease progression (in TCGA PRAD) [36] and while higher *ACADM* expression displayed a trend toward disease progression, this was not significant (*p* = 0.065, Log-Rank test with maximized cut-off groups) (Additional file 7: Figure S5C and S5D).

### Fatty acid stimulation enhances proliferation and transcription of AR responsive genes

**Fig. 7:**
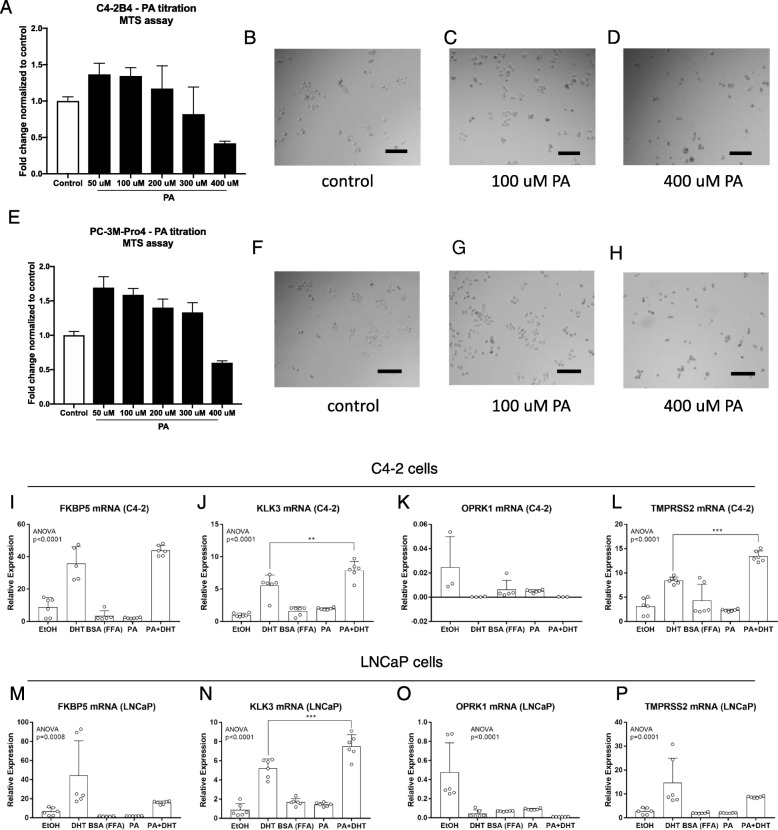
In vitro functional characterization of DHT and palmitate (PA) stimulation on AR positive and negative PCa cells. **a** Proliferation assessed in C4–2B by MTS assay upon PA (range 50–400 μM) or vehicle (control) stimulation for 48 h. **b**-**d** Representative bright field images of cultured cells under indicated experimental conditions. **e** Proliferation assessed in PC-3 M-Pro4 by MTS assay upon PA (range 50–400 μM) or vehicle (control) stimulation for 48 h. **f**-**h** Representative bright field images of cultured cells upon experimental conditions. **i**-**l** Relative expression or AR responsive genes upon dihydrotestosterone (DHT) or control (EtOH) stimulation, and palmitate (PA) or vehicle (BSA fatty acid free-FFA) or combination (PA + DHT) in AR positive C4–2 and (M-P) LNCaP cells. P-value indicated in the plots is relative to ANOVA. Multiple comparison significance between experimental condition is indicated by * (** *p* < 0.01, *** *p* < 0.001). Details related to quantification and normalization are included in the Methods section

Finally, we investigated the effect of fatty acid treatment on proliferation in C4–2B4 and PC-3 M-Pro4 cells, and regulation of androgen receptor (AR) responsive genes in AR positive LNCaP and its derivative C4–2 cells. Treatment of C4–2B4 and PC-3 M-Pro4 cells with palmitic acid (PA) (range between 50 and 200 μM) resulted in increased proliferation (Fig. 7a, b, c, d and Fig. 7e, f, g and h, respectively). Administration of PA alone in C4–2 cells was not able to induce expression of AR-responsive genes (Fig. 7i, j, k and l). However, administration of PA in combination with DHT, resulted in an additive effect tha was significant for *KLK3* and *TMPRSS2* (Fig. 7j and l). These results were confirmed in LNCaP cells (Fig. 7m, n, o, and p) where the additive effect was consistent for *KLK3* (Fig. 7n) but not for *TMPRSS2* (Fig. 7p).

## Discussion

In this study, we have provided evidence that preoperative plasma fatty acid metabolites can inform risk of prostate cancer progression. Specifically, we find that medium-chain acylcarnitines are positively associated with the risk of PSA progression and long-chain acylcarnitines are inversely associated with progression of PCa locally and to the bone. Additionally, in primary and metastatic cases, medium-chain acylcarnitines were positively associated with suberic acid, which also correlated with the risk of PSA progression. Two multi-center studies conducted by Schmidt et al. [19, 20] have also identified a correlation between acylcarnitines and PCa disease by measuring pre-diagnostic plasma metabolites and searching for those that appeared to be associated with a certain PCa grade or stage. In these studies, men with higher plasma concentrations of long-chain acylcarnitines (C18:1 and C18:2) had lower risk of advanced stage prostate cancer at diagnosis, while propionylcarntine (C3) and methionine metabolism appeared to be positively associate with aggressive disease and PCa death [19].

The main difference between these studies and our study is that Schmidt et al. [19, 20] employed pre-diagnostic plasma to identify molecules associated with risk of developing PCa, while we analyzed pre-operative-plasma, with the goal of linking specific metabolite profile with disease progression and risk stratification groups as defined by the NCCN guidelines. We found that reduced incidence of lymph node and bone progression correlated with higher levels of long-chain acylcarnitines. Despite looking at a different end points, employing a reduced sample size and starting from different samples, it is remarkable that our data are aligned with the results of Schmidt et al. [19, 20] and seem to suggest that better patient outcome is associated with elevated levels of long-chain acylcarnitines in both pre-diagnostic and pre-operative plasma.

Our extended analysis of all annotated metabolites indicates that the perturbation of the acylcarnitine metabolism in primary and metastatic cases is consistent with that of other related metabolites. For example, we detected a strong and significant positive correlation between medium-chain acylcarnitines and suberic acid, which is present in the urine of patients with fatty acid oxidation disorders and was elevated in individuals with perturbations in ACADM [51]. The fact that medium-chain acylcarnitines and suberic acid were both elevated in the same samples in this study, might reflect the metabolic state of more aggressive prostate cancers. In addition to their correlation, it is remarkable that medium-chain acylcarnitines and suberic acid both display the same inverse association with tryptophan. In a study conducted on serum from 64 PCa cases and 50 healthy controls, Zang et al. shown that tryptophan is a specific discriminatory metabolite of PCa [57]. It has been shown that cancer increases the consumption of tryptophan to evade immune control [58] and this has been linked to IDO1 activity. Degradation of L-tryptophan by IDO1 promotes CPT1 activity, thereby enhancing fatty acid metabolism [54]. In PCa, PTEN-deficient tumors have been associated with an immunosuppressive microenvironment mediated by increased expression of IDO1 [59].

The patterns of metabolites that we observed in primary versus metastatic samples and the transcriptional analysis of tumor tissue versus normal tissue, are consistent with the elevated levels of *IDO1* that we found in metastatic compared with primary cases and that we found in androgen-ablation resistant PCa metastatic samples [55]. Increased levels of IDO1 in patient with metastatic disease compared with patients with primary tumors would lead to a strong reduction in L-tryptohpan and a corresponding increase in fatty acid metabolism products (C2 and medium-chain acylcarnitines), as we observed in our cohort. However, the precise association with ACADM activity should be further investigated, since its increased levels in metastatic versus primary cases might t also suggest a decrease in the products of the enzymatic reactions (i.e. medium-chain acylcarnitines), rather than an accumulation, as documented here.

Levels of other products of tryptophan metabolism support the link between tryptophan and medium-chain acylcarnitines. In a metastatic setting, a reduction of L-tryptophan levels due to high *IDO1* expression, would lead to reduced levels of indoleacetaldehyde, which is a substrate of ALDH7A1. Further experimentation should be conducted to elucidate whether the augmented levels of ALDH7A1, already reported to be a marker of advanced PCa [52, 53], can be supported by these metabolic alterations and eventually represent an adaptation mechanism resulting in higher levels of indoleacetic acid, thus supporting its correlation with PSA and lymph node progression.

In addition to correlating fatty acid metabolism with risk or PCa progression, we have also shown that fatty acids impact PCa cell behavior. We find that PA and DHT have an additive effect on the expression of AR responsive genes in LNCaP and C4–2 cells, which normally grow in medium without DHT supplementation and are therefore capable of proliferating under castration conditions. These data are in line with recent findings that interference in fatty acid metabolism via inhibition of de novo lipogenesis targets androgen receptor signaling in castration-resistant prostate cancer [60]. Additionally, they reinforce previous data that PCa cell lines have fatty acid and fatty acid β-oxidation altered [61]. Moreover, these observations are supported by the increase in proliferation that we observed upon PA stimulation of PC-3 M-Pro4 cells, which lack AR expression and are by definition a castration resistant model. While our observations are supported by recent evidence that suppressing fatty acid uptake has therapeutic effects in preclinical models of PCa [62], additional experimentation should be conducted to elucidate whether increased fatty acid levels affect AR signaling to promote an aggressive phenotype.

While the number of patients allocated in each of the classification groups (low risk, high risk without progression and high risk with progression) is quite small, the total number of PCa cases (*n* = 33) is in line with other studies conducted in single centers [17, 18, 57, 63, 64] and, importantly, includes age-matched primary cases. Our results are primarily associated with disease progression rather than disease status and the fact that our control group displayed a significantly lower age at blood draw compared to PCa cases (primary and metastatic), does not impact with the association that we conduct within the group of primary cases. Furthermore, it should be noted that this is a pilot study, and our findings are confirmatory of previously published work, as elaborated in this discussion. The principal discriminative factor between the groups of our primary cases was the progression to lymph node or bone metastasis. However, even with this extreme end point, we were not able to identify metabolites specific for a particular risk group. Tissue samples from the PCa cases included in this study cohort are currently being retrieved to further validate the metabolic association with stratified risk groups.

## Conclusions

In conclusion, our pilot study confirms and extends previous findings that fatty acid metabolism is associated with advanced PCa and risk of disease progression and our results suggest that evaluation of acylcarnitines profile might contribute to improve patient stratification. Our findings further demonstrate that analysis of metabolites in plasma is a valid method for generating novel hypothesis worth to be validated in an independent cohort and to identify patients who are at risk of disease progression. Finally, our data suggest that this metabolomics strategy could be employed to assist patient stratification at diagnosis.

## Supplementary information

**Additional file 1: Table S1.** List of sequences for primers used for RT-qPCR.

**Additional file 2: Figure S1.** Classification of controls and cases by principal component analysis **A)** Principal component analysis (PCA) of all the m/z features measured in positive and negative ionization mode allocated for controls and cases. The variation retained by PC1 (16.3%) is represented of the X axis and the variation retained by the PC2 (9.3%) is represented on the Y axis. Ellipses represent the 95% confidence interval for each group. **B)** Principal component analysis (PCA) of the annotated m/z features in positive and negative ionization mode allocated for controls and cases. The variation retained by PC1 (16.7%) is represented of the X axis and the variation retained by the PC2 (13.5%) is represented on the Y axis. Ellipses represent the 95% confidence interval for each group.

**Additional file 3: Figure S2.** Hazard ratio and 95% confidence interval by median for the association of short-, medium- and long-chain aylcarnitines with disease progression. **A)** Hazard ratios and 95% confidence interval (CI) for the association of short-, (Short_med), medium (Medium_med) and long-chain Acetylcarnitines (Long_med) with the risk of PSA progression, **(B)** local progression, **(C)** lymph node progression and **(D)** bone progression. Groups (lower and higher risk) were separate by median cut-off (_med suffix) of the normalized abundances for each class of the molecules.

**Additional file 4: Figure S3.** Correlation matrix for all the identified metabolites in metastatic cases. Insert represents the correlation among acylcarnitines and all the annotated metabolites in metastatic cases. The sizes of the circles are dependent on the Pearson correlation coefficient. Blue circles correspond to positive correlations and red circles correspond to negative correlations. Insignificant correlation (*p* > 0.05) are indicated by an empty square box.

**Additional file 5: Table S2.** List of LC-MS characteristics (adduct, retention time error, mass error, isotope similarity and best-matched fragment) of annotated ions of interest measured in serum by non-targeted Q-TOF analysis in ESI+ and ESI- mode.

**Additional file 6: Figure S4.** Illustration of *IDO1* and *ACADM* expression in primary and metastatic samples and tumor and tumor-adjacent tissues. *IDO1* expression data of primary and tissue specific androgen ablation resistant metastasis from GSE6752 **(A)** and tumor versus tumor adjacent tissues from GSE6919 **(B)**. *ACADM* expression data in the same set of samples are displayed in **(C-D)**. Fold change (FC) is calculated versus the normal or primary tumor samples and *p*-value (P) for significance between two groups estimated by t-test or ANOVA between more groups.

**Additional file 7: Figure S5**. Illustration of IDO1 and ACADM genomic alterations and correlation with tumor progression in TCGA PRAD data. **A-B)** Outlook of IDO1 and ACADM genomic alterations in publically available prostate cancer related TCGA datasets. Alteration frequencies related to mutation (green), fusion (purple), amplification (red) and deep deletion (blue) are represented on the Y axis. Data retrieved from cBioPortal. **C-D)** Kaplan Meier curves for tumor progression-free survival from TCGA PRAD data retrieved from Firehose. Survival was estimated by calculation of optimal cut points and significance estimated by log-rank test. Blue solid line corresponds to high expression group, red dotted line corresponds to low expression group. See Methods section for statistic details.

## Data Availability

All data generated during this study are included in this published article [and its additional files]. The datasets analyzed during the current study are available in the Gene Expression Omnibus repository [https://www.ncbi.nlm.nih.gov/geo/] under accession number GSE6752 and GSE6919. The TCGA PRAD data are available in the Firehose Broad GDAC repository [http://gdac.broadinstitute.org/].

## Abbreviations

ACADM: Medium-chain Acyl-CoA Dehydrogenase
ALDH7A1: Aldehyde dehydrogenase 7 family member A1
AR: Androgen receptor
BPH: Benign prostatic hyperplasia
CPT1: Carnitine palmitoyltransferase I
DHT: Dihydrotestosterone
FA: Fatty acids
HMDB: Human Metabolome Database
HMRS: High-resolution mass spectrometry
HR: Hazard ratio
IDO1: Indoleamine 2,3-dioxygenase
NCCN: National comprehensive cancer network
PA: Palmitic acid
PCa: Prostate cancer
PSA: Prostate specific antigen
UHPLC: Ultra-high performance liquid chromatography

## References

[1] Bray F, Ferlay J, Soerjomataram I, Siegel RL, Torre LA, Jemal A (2018). Global cancer statistics 2018: GLOBOCAN estimates of incidence and mortality worldwide for 36 cancers in 185 countries. CA Cancer J Clin.

[2] Spahn M, Boxler S, Joniau S, Moschini M, Tombal B, Karnes RJ (2015). What is the need for prostatic biomarkers in prostate Cancer management?. Curr Urol Rep.

[3] Ramautar R, Berger R, van der Greef J, Hankemeier T (2013). Human metabolomics: strategies to understand biology. Curr Opin Chem Biol.

[4] Ferro M, Buonerba C, Terracciano D, Lucarelli G, Cosimato V, Bottero D, Deliu VM, Ditonno P, Perdona S, Autorino R (2016). Biomarkers in localized prostate cancer. Future Oncol.

[5] Johnson CH, Gonzalez FJ (2012). Challenges and opportunities of metabolomics. J Cell Physiol.

[6] Giunchi F, Fiorentino M, Loda M (2019). The metabolic landscape of prostate Cancer. Eur Urol Oncol.

[7] Trock BJ (2011). Application of metabolomics to prostate cancer. Urol Oncol.

[8] Lima AR, Bastos Mde L, Carvalho M, Guedes de Pinho P (2016). biomarker discovery in human prostate Cancer: an update in metabolomics studies. Transl Oncol.

[9] Ferro M, Terracciano D, Buonerba C, Lucarelli G, Bottero D, Perdona S, Autorino R, Serino A, Cantiello F, Damiano R (2017). The emerging role of obesity, diet and lipid metabolism in prostate cancer. Future Oncol.

[10] Wu X, Daniels G, Lee P, Monaco ME (2014). Lipid metabolism in prostate cancer. Am J Clin Exp Urol.

[11] Warburg O, Wind F, Negelein E (1927). The metabolism of tumors in the body. J Gen Physiol.

[12] Zadra G, Photopoulos C, Loda M (2013). The fat side of prostate cancer. Biochim Biophys Acta.

[13] Liu Y, Zuckier LS, Ghesani NV (2010). Dominant uptake of fatty acid over glucose by prostate cells: a potential new diagnostic and therapeutic approach. Anticancer Res.

[14] Liu Y (2006). Fatty acid oxidation is a dominant bioenergetic pathway in prostate cancer. Prostate Cancer Prostatic Dis.

[15] Grundmark B, Garmo H, Loda M, Busch C, Holmberg L, Zethelius B (2010). The metabolic syndrome and the risk of prostate cancer under competing risks of death from other causes. Cancer Epidemiol Biomark Prev.

[16] de Cobelli O, Terracciano D, Tagliabue E, Raimondi S, Galasso G, Cioffi A, Cordima G, Musi G, Damiano R, Cantiello F (2015). Body mass index was associated with upstaging and upgrading in patients with low-risk prostate cancer who met the inclusion criteria for active surveillance. Urol Oncol.

[17] Thysell E, Surowiec I, Hornberg E, Crnalic S, Widmark A, Johansson AI, Stattin P, Bergh A, Moritz T, Antti H (2010). Metabolomic characterization of human prostate cancer bone metastases reveals increased levels of cholesterol. PLoS One.

[18] Giskeodegard GF, Hansen AF, Bertilsson H, Gonzalez SV, Kristiansen KA, Bruheim P, Mjos SA, Angelsen A, Bathen TF, Tessem MB (2015). Metabolic markers in blood can separate prostate cancer from benign prostatic hyperplasia. Br J Cancer.

[19] Schmidt JA, Fensom GK, Rinaldi S, Scalbert A, Appleby PN, Achaintre D, Gicquiau A, Gunter MJ, Ferrari P, Kaaks R (2017). Pre-diagnostic metabolite concentrations and prostate cancer risk in 1077 cases and 1077 matched controls in the European prospective investigation into Cancer and nutrition. BMC Med.

[20] Schmidt Julie A., Fensom Georgina K., Rinaldi Sabina, Scalbert Augustin, Appleby Paul N., Achaintre David, Gicquiau Audrey, Gunter Marc J., Ferrari Pietro, Kaaks Rudolf, Kühn Tilman, Boeing Heiner, Trichopoulou Antonia, Karakatsani Anna, Peppa Eleni, Palli Domenico, Sieri Sabina, Tumino Rosario, Bueno‐de‐Mesquita Bas, Agudo Antonio, Sánchez Maria‐Jose, Chirlaque María‐Dolores, Ardanaz Eva, Larrañaga Nerea, Perez‐Cornago Aurora, Assi Nada, Riboli Elio, Tsilidis Konstantinos K., Key Timothy J., Travis Ruth C. (2019). Patterns in metabolite profile are associated with risk of more aggressive prostate cancer: A prospective study of 3,057 matched case–control sets from EPIC. International Journal of Cancer.

[21] Crowe FL, Allen NE, Appleby PN, Overvad K, Aardestrup IV, Johnsen NF, Tjonneland A, Linseisen J, Kaaks R, Boeing H (2008). Fatty acid composition of plasma phospholipids and risk of prostate cancer in a case-control analysis nested within the European prospective investigation into Cancer and nutrition. Am J Clin Nutr.

[22] Carroll PR, Parsons JK, Andriole G, Bahnson RR, Castle EP, Catalona WJ, Dahl DM, Davis JW, Epstein JI, Etzioni RB (2016). NCCN guidelines insights: prostate Cancer early detection, version 2.2016. J Natl Compr Cancer Netw.

[23] Tuck MK, Chan DW, Chia D, Godwin AK, Grizzle WE, Krueger KE, Rom W, Sanda M, Sorbara L, Stass S (2009). Standard operating procedures for serum and plasma collection: early detection research network consensus statement standard operating procedure integration working group. J Proteome Res.

[24] Mohler J, Bahnson RR, Boston B, Busby JE, D'Amico A, Eastham JA, Enke CA, George D, Horwitz EM, Huben RP (2010). NCCN clinical practice guidelines in oncology: prostate cancer. J Natl Compr Cancer Netw.

[25] Mohler JL, Armstrong AJ, Bahnson RR, D'Amico AV, Davis BJ, Eastham JA, Enke CA, Farrington TA, Higano CS, Horwitz EM (2016). Prostate Cancer, version 1.2016. J Natl Compr Cancer Netw.

[26] Rindlisbacher B, Schmid C, Geiser T, Bovet C, Funke-Chambour M (2018). Serum metabolic profiling identified a distinct metabolic signature in patients with idiopathic pulmonary fibrosis - a potential biomarker role for LysoPC. Respir Res.

[27] Wishart DS, Tzur D, Knox C, Eisner R, Guo AC, Young N, Cheng D, Jewell K, Arndt D, Sawhney S (2007). HMDB: the human Metabolome database. Nucleic Acids Res.

[28] Therneau T: A Package for Survival Analysis in S. In*.*, 2.38 edn; 2015.

[29] Terry M (2000). Therneau PMG: modeling survival data: extending the cox model. In.

[30] Alboukadel Kassambara MK: survminer: Drawing Survival Curves using 'ggplot2'. In*.*, 0.4.3 edn; 2018.

[31] SCHOENFELD DAVID (1982). Partial residuals for the proportional hazards regression model. Biometrika.

[32] Frank E Harrell Jr: **Hmisc:** Harrell Miscellaneous. In*.*, 4.2–0 edn; 2019.

[33] Dumas J, Gargano MA, Dancik GM (2016). shinyGEO: a web-based application for analyzing gene expression omnibus datasets. Bioinformatics.

[34] Cerami E, Gao J, Dogrusoz U, Gross BE, Sumer SO, Aksoy BA, Jacobsen A, Byrne CJ, Heuer ML, Larsson E (2012). The cBio cancer genomics portal: an open platform for exploring multidimensional cancer genomics data. Cancer Discov.

[35] Gao J., Aksoy B. A., Dogrusoz U., Dresdner G., Gross B., Sumer S. O., Sun Y., Jacobsen A., Sinha R., Larsson E., Cerami E., Sander C., Schultz N. (2013). Integrative Analysis of Complex Cancer Genomics and Clinical Profiles Using the cBioPortal. Science Signaling.

[36] Broad Institute TCGA Genome Data Analysis Center (2016): Analysis Overview for Prostate Adenocarcinoma (Primary solid tumor cohort) - 28 January 2016. In*.*: Broad Institute of MIT and Harvard; 2016.

[37] Samur MK (2014). RTCGAToolbox: a new tool for exporting TCGA Firehose data. PLoS One.

[38] Sebastien Le JJ, Francois Husson,: FactoMineR: An R Package for Multivariate Analysis. Journal of Statistical Software. In*.*, 25(1), 1–18 edn; 2008.

[39] Wickham H (2016). ggplot2: elegant graphics for data analysis. In.

[40] RStudio Team: RStudio: integrated development for R. RStudio. In*.* Boston, MA: Inc.; 2016.

[41] R Core Team: R: a language and environment for statistical computing. In*.* Vienna, Austria: R Foundation for Statistical Computing; 2019.

[42] Pettaway CA, Pathak S, Greene G, Ramirez E, Wilson MR, Killion JJ, Fidler IJ (1996). Selection of highly metastatic variants of different human prostatic carcinomas using orthotopic implantation in nude mice. Clin Cancer Res.

[43] Wu HC, Hsieh JT, Gleave ME, Brown NM, Pathak S, Chung LW (1994). Derivation of androgen-independent human LNCaP prostatic cancer cell sublines: role of bone stromal cells. Int J Cancer.

[44] Thalmann GN, Anezinis PE, Chang SM, Zhau HE, Kim EE, Hopwood VL, Pathak S, von Eschenbach AC, Chung LW (1994). Androgen-independent cancer progression and bone metastasis in the LNCaP model of human prostate cancer. Cancer Res.

[45] Horoszewicz JS, Leong SS, Kawinski E, Karr JP, Rosenthal H, Chu TM, Mirand EA, Murphy GP (1983). LNCaP model of human prostatic carcinoma. Cancer Res.

[46] Horoszewicz JS, Leong SS, Chu TM, Wajsman ZL, Friedman M, Papsidero L, Kim U, Chai LS, Kakati S, Arya SK (1980). The LNCaP cell line--a new model for studies on human prostatic carcinoma. Prog Clin Biol Res.

[47] Kiener M, Chen L, Krebs M, Grosjean J, Klima I, Kalogirou C, Riedmiller H, Kneitz B, Thalmann GN, Snaar-Jagalska E (2019). miR-221-5p regulates proliferation and migration in human prostate cancer cells and reduces tumor growth in vivo. BMC Cancer.

[48] Zoni E, Astrologo L, Ng CKY, Piscuoglio S, Melsen J, Grosjean J, Klima I, Chen L, Snaar-Jagalska EB, Flanagan K (2019). Therapeutic targeting of CD146/MCAM reduces bone metastasis in prostate Cancer. Mol Cancer Res.

[49] Niederberger P, Farine E, Arnold M, Wyss RK, Sanz MN, Mendez-Carmona N, Gahl B, Fiedler GM, Carrel TP, Tevaearai Stahel HT (2017). High pre-ischemic fatty acid levels decrease cardiac recovery in an isolated rat heart model of donation after circulatory death. Metabolism.

[50] Schlaepfer IR, Rider L, Rodrigues LU, Gijon MA, Pac CT, Romero L, Cimic A, Sirintrapun SJ, Glode LM, Eckel RH (2014). Lipid catabolism via CPT1 as a therapeutic target for prostate cancer. Mol Cancer Ther.

[51] Hagen T, Korson MS, Sakamoto M, Evans JE (1999). A GC/MS/MS screening method for multiple organic acidemias from urine specimens. Clin Chim Acta.

[52] van den Hoogen C, van der Horst G, Cheung H, Buijs JT, Pelger RC, van der Pluijm G (2011). The aldehyde dehydrogenase enzyme 7A1 is functionally involved in prostate cancer bone metastasis. Clin Exp Metastasis.

[53] van den Hoogen C, van der Horst G, Cheung H, Buijs JT, Lippitt JM, Guzman-Ramirez N, Hamdy FC, Eaton CL, Thalmann GN, Cecchini MG (2010). High aldehyde dehydrogenase activity identifies tumor-initiating and metastasis-initiating cells in human prostate cancer. Cancer Res.

[54] Eleftheriadis T, Pissas G, Sounidaki M, Tsogka K, Antoniadis N, Antoniadi G, Liakopoulos V, Stefanidis I (2016). Indoleamine 2,3-dioxygenase, by degrading L-tryptophan, enhances carnitine palmitoyltransferase I activity and fatty acid oxidation, and exerts fatty acid-dependent effects in human alloreactive CD4+ T-cells. Int J Mol Med.

[55] Chandran UR, Ma C, Dhir R, Bisceglia M, Lyons-Weiler M, Liang W, Michalopoulos G, Becich M, Monzon FA (2007). Gene expression profiles of prostate cancer reveal involvement of multiple molecular pathways in the metastatic process. BMC Cancer.

[56] Yu YP, Landsittel D, Jing L, Nelson J, Ren B, Liu L, McDonald C, Thomas R, Dhir R, Finkelstein S (2004). Gene expression alterations in prostate cancer predicting tumor aggression and preceding development of malignancy. J Clin Oncol.

[57] Zang X, Jones CM, Long TQ, Monge ME, Zhou M, Walker LD, Mezencev R, Gray A, McDonald JF, Fernandez FM (2014). Feasibility of detecting prostate cancer by ultraperformance liquid chromatography-mass spectrometry serum metabolomics. J Proteome Res.

[58] Prendergast GC (2011). Cancer: why tumours eat tryptophan. Nature.

[59] Vidotto T, Saggioro FP, Jamaspishvili T, Chesca DL, Picanco de Albuquerque CG, Reis RB, Graham CH, Berman DM, Siemens DR, squire JA (2019). PTEN-deficient prostate cancer is associated with an immunosuppressive tumor microenvironment mediated by increased expression of IDO1 and infiltrating FoxP3+ T regulatory cells. Prostate.

[60] Zadra G, Ribeiro CF, Chetta P, Ho Y, Cacciatore S, Gao X, Syamala S, Bango C, Photopoulos C, Huang Y (2019). Inhibition of de novo lipogenesis targets androgen receptor signaling in castration-resistant prostate cancer. Proc Natl Acad Sci U S A.

[61] Lima AR, Araujo AM, Pinto J, Jeronimo C, Henrique R, Bastos ML, Carvalho M, Guedes de Pinho P (2018). discrimination between the human prostate normal and cancer cell exometabolome by GC-MS. Sci Rep.

[62] Watt Matthew J., Clark Ashlee K., Selth Luke A., Haynes Vanessa R., Lister Natalie, Rebello Richard, Porter Laura H., Niranjan Birunthi, Whitby Sarah T., Lo Jennifer, Huang Cheng, Schittenhelm Ralf B., Anderson Kimberley E., Furic Luc, Wijayaratne Poornima R., Matzaris Maria, Montgomery Magdalene K., Papargiris Melissa, Norden Sam, Febbraio Maria, Risbridger Gail P., Frydenberg Mark, Nomura Daniel K., Taylor Renea A. (2019). Suppressing fatty acid uptake has therapeutic effects in preclinical models of prostate cancer. Science Translational Medicine.

[63] Stabler S, Koyama T, Zhao Z, Martinez-Ferrer M, Allen RH, Luka Z, Loukachevitch LV, Clark PE, Wagner C, Bhowmick NA (2011). Serum methionine metabolites are risk factors for metastatic prostate cancer progression. PLoS One.

[64] Saylor PJ, Karoly ED, Smith MR (2012). Prospective study of changes in the metabolomic profiles of men during their first three months of androgen deprivation therapy for prostate cancer. Clin Cancer Res.

