# Supplementary figures and images for: Preoperative plasma fatty acid metabolites inform risk of prostate cancer progression and may be used for personalized patient stratification
Source: BMC Cancer. 2019 Dec 16;19:1216. doi: 10.1186/s12885-019-6418-2 (PMC6916032; doi:10.1186/s12885-019-6418-2)

# Suppl. Figure 1

A

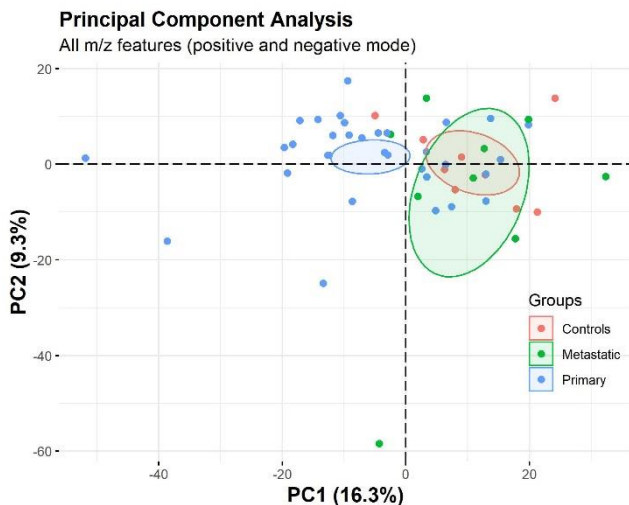

B

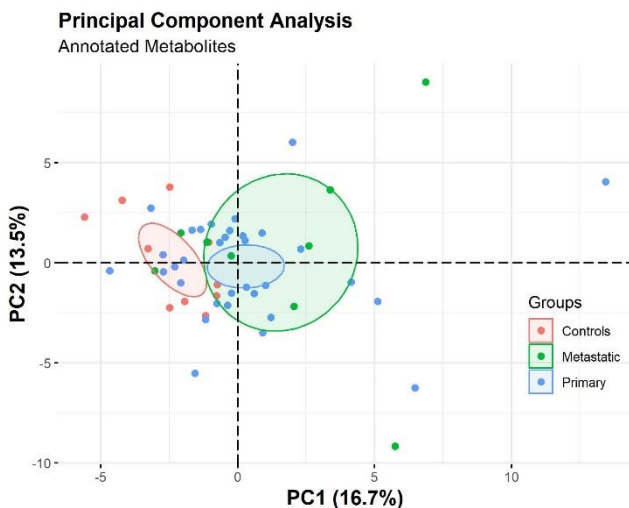

Supplement: Supplementary file 2 — Additional file 2: Figure S1. Classification of controls and cases by principal component analysis A) Principal component analysis (PCA) of all the m/z features measured in positive and negative ionization mode allocated for controls and cases. The variation retained by PC1 (16.3%) is represented of the X axis and the variation retained by the PC2 (9.3%) is represented on the Y axis. Ellipses represent the 95% confidence interval for each group. B) Principal component analysis (PCA) of the annotated m/z features in positive and negative ionization mode allocated for controls and cases. The variation retained by PC1 (16.7%) is represented of the X axis and the variation retained by the PC2 (13.5%) is represented on the Y axis. Ellipses represent the 95% confidence interval for each group. [file 12885_2019_6418_MOESM2_ESM.pdf]

# Supplementary Figure 3

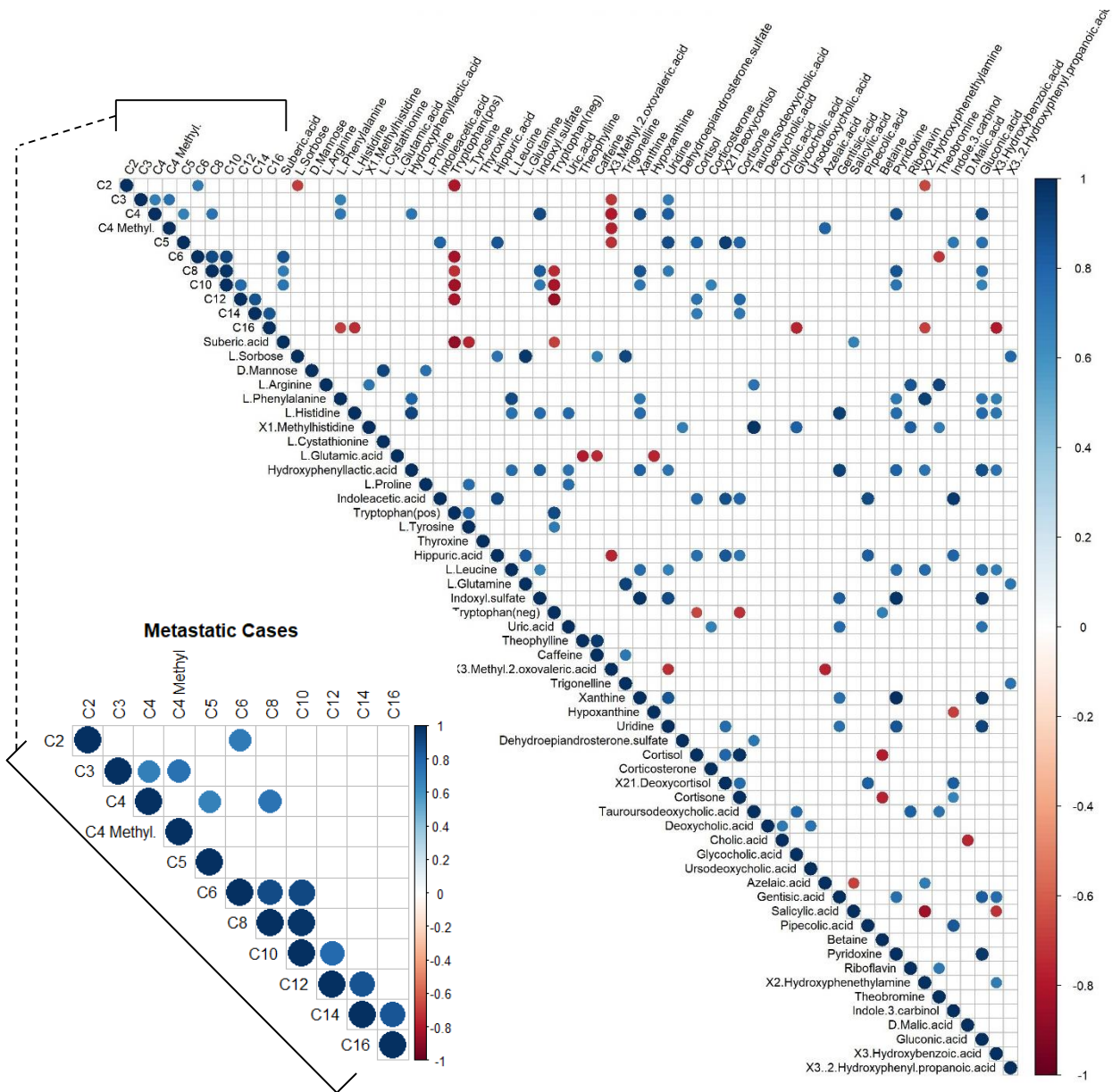

Supplement: Supplementary file 4 — Additional file 4: Figure S3. Correlation matrix for all the identified metabolites in metastatic cases. Insert represents the correlation among acylcarnitines and all the annotated metabolites in metastatic cases. The sizes of the circles are dependent on the Pearson correlation coefficient. Blue circles correspond to positive correlations and red circles correspond to negative correlations. Insignificant correlation (p > 0.05) are indicated by an empty square box. [file 12885_2019_6418_MOESM4_ESM.pdf]

# Supplementary Figure 4

A

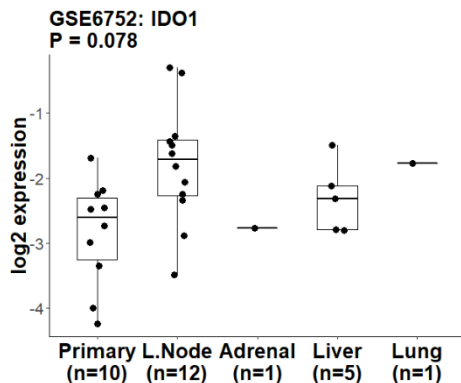

B

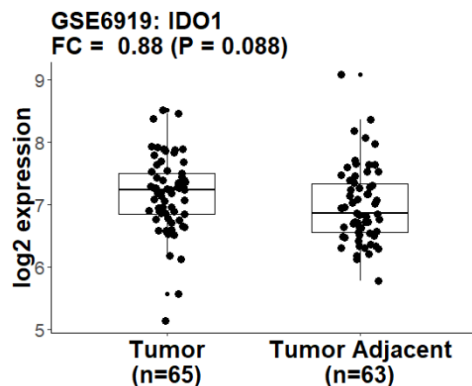

C

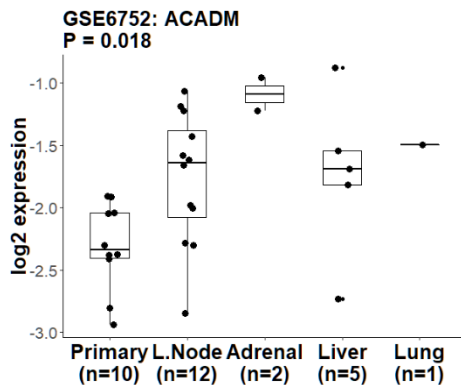

D

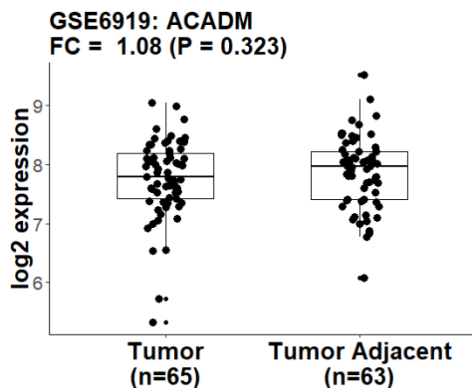

Supplement: Supplementary file 6 — Additional file 6: Figure S4. Illustration of IDO1 and ACADM expression in primary and metastatic samples and tumor and tumor-adjacent tissues. IDO1 expression data of primary and tissue specific androgen ablation resistant metastasis from GSE6752 (A) and tumor versus tumor adjacent tissues from GSE6919 (B). ACADM expression data in the same set of samples are displayed in (C-D). Fold change (FC) is calculated versus the normal or primary tumor samples and p-value (P) for significance between two groups estimated by t-test or ANOVA between more groups. [file 12885_2019_6418_MOESM6_ESM.pdf]

# Supplementary Figure 5

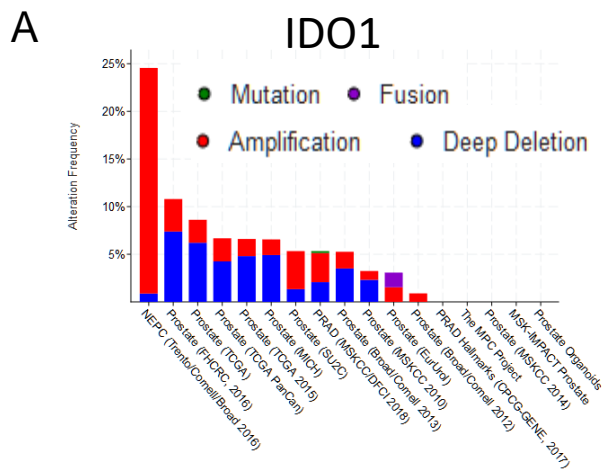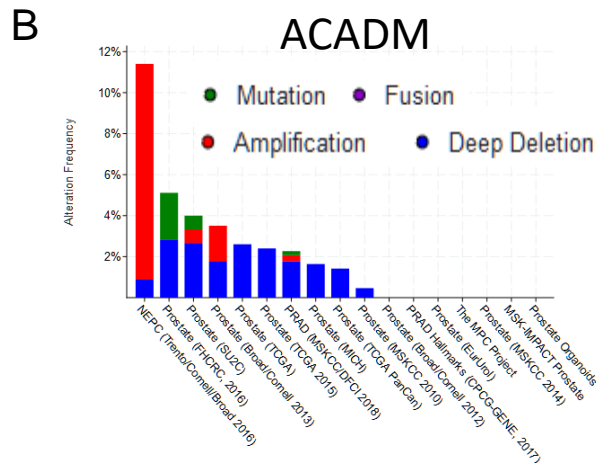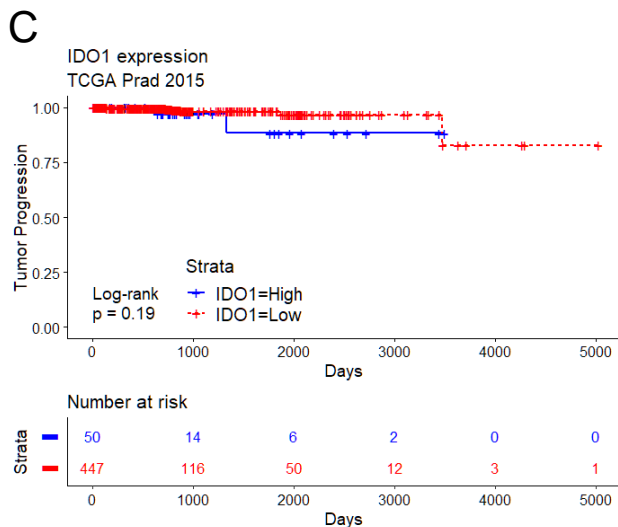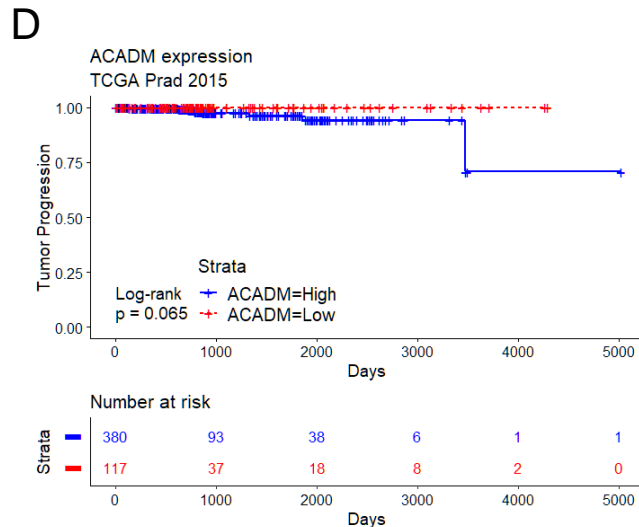

Supplement: Supplementary file 7 — Additional file 7: Figure S5. Illustration of IDO1 and ACADM genomic alterations and correlation with tumor progression in TCGA PRAD data. A-B) Outlook of IDO1 and ACADM genomic alterations in publically available prostate cancer related TCGA datasets. Alteration frequencies related to mutation (green), fusion (purple), amplification (red) and deep deletion (blue) are represented on the Y axis. Data retrieved from cBioPortal. C-D) Kaplan Meier curves for tumor progression-free survival from TCGA PRAD data retrieved from Firehose. Survival was estimated by calculation of optimal cut points and significance estimated by log-rank test. Blue solid line corresponds to high expression group, red dotted line corresponds to low expression group. See Methods section for statistic details. [file 12885_2019_6418_MOESM7_ESM.pdf]
