# Supplementary material for: Preoperative plasma fatty acid metabolites inform risk of prostate cancer progression and may be used for personalized patient stratification
Source: BMC Cancer. 2019 Dec 16;19:1216. doi: 10.1186/s12885-019-6418-2 (PMC6916032; doi:10.1186/s12885-019-6418-2)

Supplementary Figure 2

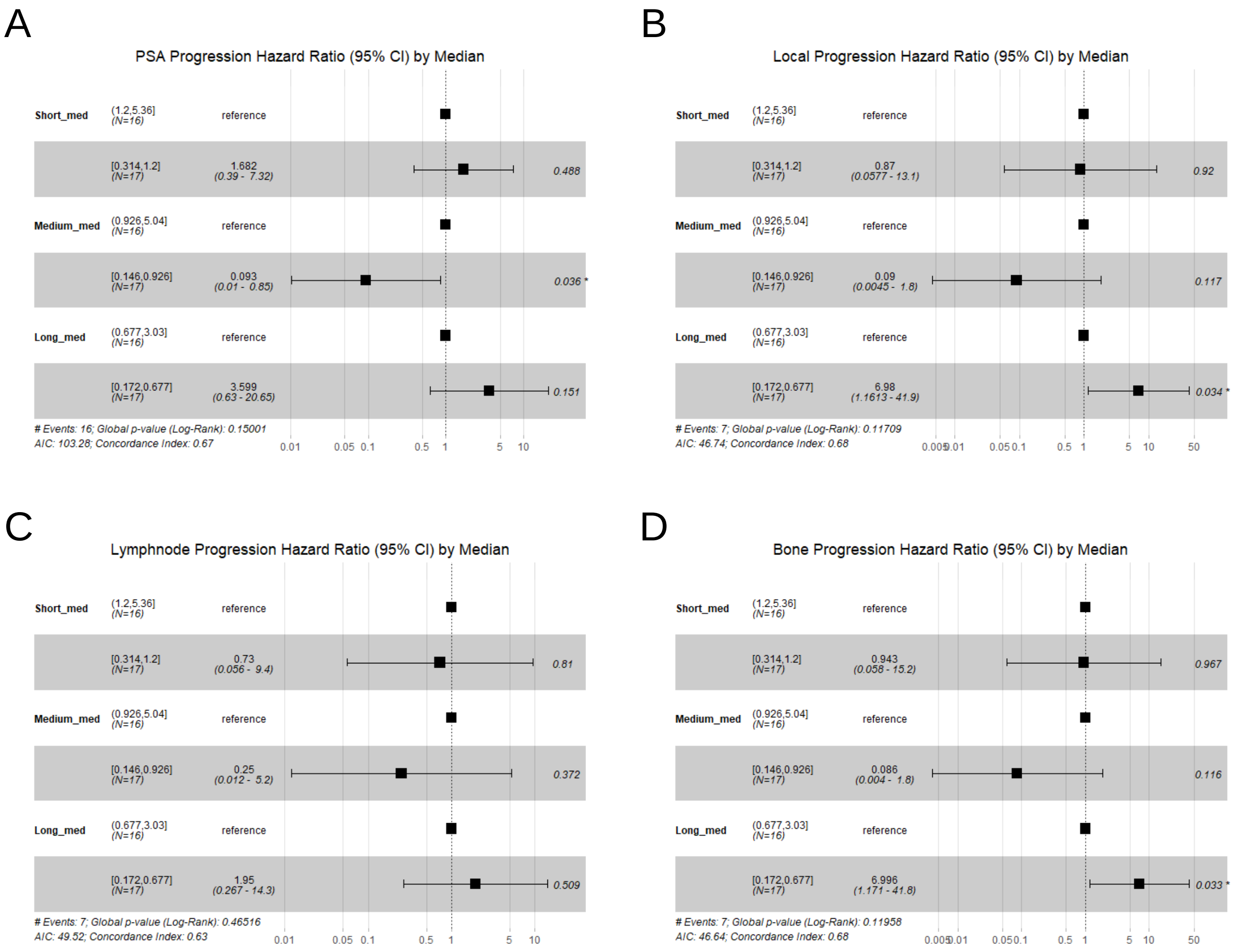

Supplement: Supplementary file 3 — Additional file 3: Figure S2. Hazard ratio and 95% confidence interval by median for the association of short-, medium- and long-chain aylcarnitines with disease progression. A) Hazard ratios and 95% confidence interval (CI) for the association of short-, (Short_med), medium (Medium_med) and long-chain Acetylcarnitines (Long_med) with the risk of PSA progression, (B) local progression, (C) lymph node progression and (D) bone progression. Groups (lower and higher risk) were separate by median cut-off (_med suffix) of the normalized abundances for each class of the molecules. [file 12885_2019_6418_MOESM3_ESM.pdf]
